# Daphnane Diterpenoids from *Trigonostemon lii* and Inhibition Activities Against HIV-1

**DOI:** 10.1007/s13659-020-00231-7

**Published:** 2020-02-11

**Authors:** Cheng-Jian Tan, Shi-Fei Li, Ning Huang, Yu Zhang, Ying-Tong Di, Yong-Tang Zheng, Xiao-Jiang Hao

**Affiliations:** 1 State Key Laboratory of Phytochemistry and Plant Resources in West China, Kunming Institute of Botany, Chinese Academy of Sciences, Kunming, 650204 Yunnan People’s Republic of China; 2 Key Laboratory of Bioactive Peptides of Yunnan Province/Key Laboratory of Animal Models and Human Disease Mechanisms of the Chinese Academy of Sciences, Center for Biosafety Mega-Science, Kunming Institute of Zoology, Chinese Academy of Sciences, Kunming, 650223 Yunnan People’s Republic of China; 3 School of Ethnic Medicine, Guizhou Minzu University, Guiyang, 550025 Guizhou People’s Republic of China; 4 Institute of Molecular Science, Shanxi University, Taiyuan, 030006 Shanxi People’s Republic of China; 5 School of Basic Medical, Kunming Medical University, Kunming, 650500 Yunnan People’s Republic of China

**Keywords:** *Trigonostemon lii*, Daphnane diterpenoid, Trigonolactone, Anti-HIV

## Abstract

**Abstract:**

Natural products are the important source for the discovery of more potent anti-HIV agents. In this study, six daphnane diterpenoids including three unreported structures were isolated from *Trigonostemon lii*, which showed significant activities against HIV-1 strains replication in the nanomolar/picomolar range. Meanwhile, these diterpenoids significantly inhibited the fusion of H9/HIV-1 IIIB cells with uninfected C8166 cells, with the EC_50s_ from 1.06 to 8.73 ng/mL, and did not show any inhibition activities against HIV-1 reverse transcriptase. Moreover, all of the diterpenoids shows significant inhibitions against T20-resistan HIV-1 strains, PNL4-3gp41(36G)V38E, N42S and pNL4-3gp41(36G)V38A, N42T. The results revealed that the six diterpenoids could be a new type of potential lead candidate as an HIV entry inhibitor, particularly for those infected by T20-resistant variants.

**Graphic Abstract:**

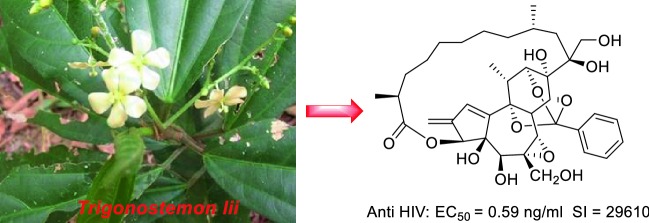

**Electronic supplementary material:**

The online version of this article (10.1007/s13659-020-00231-7) contains supplementary material, which is available to authorized users.

## Introduction

According to the latest report on the global AIDS epidemic, infections with the human immunodeficiency virus 1 (HIV-1) remain a global threat to public health [1]. Drug treatment for controlling viral loads and for prolonging patients’ lives is the main therapy for HIV-1 infections. Highly active anti-retroviral therapy now has shown significant synergistic effects on prolonging the lifetime and decreasing the mortality of patients, the issues such as toxicity, viral reservoirs and drug resistance have led to a subsequent crisis in the management of HIV/AIDS patients [2]. The potent drugs may fail at a later stage. In the light of these considerations, new classes of drugs against HIV are in urgent need to develop.

The process of HIV-1 entry into host cells considerable potential for therapeutic intervention, with viral entry proceeding through multiple sequential steps involving attachment, coreceptor blinding, and fusion [3]. The discovery and characterization of new anti-HIV agents of those steps to be the host remain priority.

**Fig. 1:**
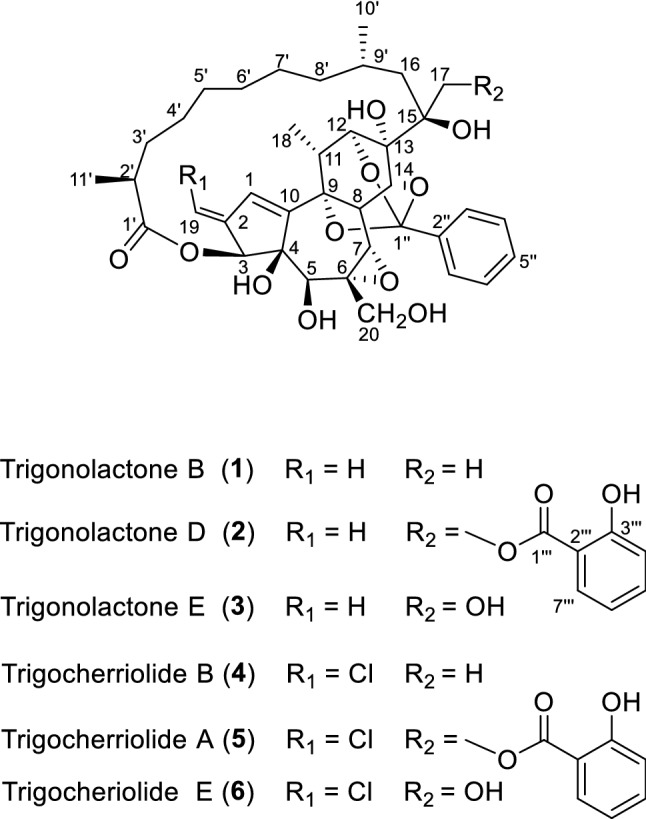
The structures of compounds **1**–**6** from the *Trigonostemon lii*

Natural products have served as the important leads for the discovery of more potent anti-HIV agents [4, 5]. However, very few natural products were discovered as having anti-HIV-1 potential in the nanomolar/picomolar range [4, 6]. Here, we reported six daphnane diterpenoids (compounds **1**–**6**) including three unreported ones [trigonolactones B (**1**), D (**2**) and E (**3**)] with significant anti-HIV-1 activity from *Trigonostemon lii* [7] (Fig. 1). These compounds not only exhibited strong inhibition on HIV replication with EC_50_ values of 0.59–8.22 ng/mL and SI values of 1811–29,610, but also displayed significantly inhibited the fusion of H9/HIV-1IIIB cells with uninfected C8166 cells with EC_50_ values of 1.06–8.73 ng/mL, while did not show any inhibition activity against HIV-1 reverse transcriptase. More important, these compounds still displayed significant inhibitions against T20-resistant HIV-1 strains, pNL4-3gp41(36G)V38E, N42S and pNL4-3gp41(36G)V38A, N42T. The structures of these compounds were established by spectroscopic approach including 1D, 2D NMR and HRMS technology.

## Results and Discussion

### Structure Elucidation

**Fig. 2:**
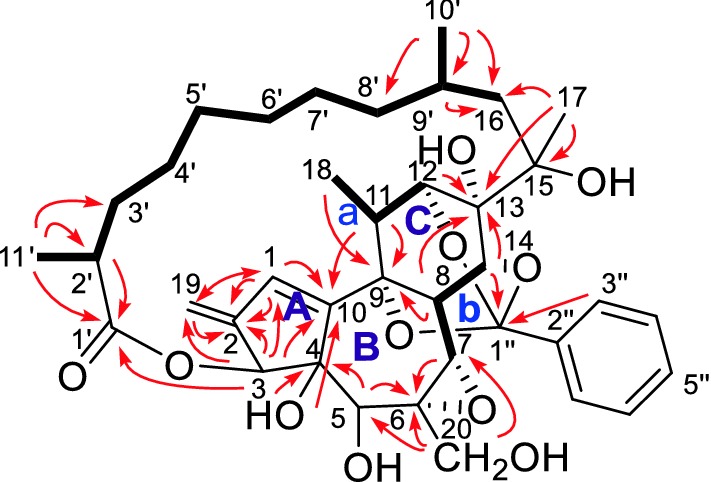
Partial fragments (**a**-**c**), ^1^H, ^1^H COSY (  ) and key HMBC ($\to$) of **1**

*Trigonolactone* B (**1**) was obtained as an optically active white powder, and the molecular formula was established to be C_38_H_50_O_11_ by HR-ESI-MS [m/z 705.3248 (M+Na)^+^, calcd 705.3250], requiring 14 degrees of unsaturation. The IR spectrum showed absorptions that were typical of hydroxy (3442 cm^−1^) and ester (1710 cm^−1^) functionalities. The ^1^H NMR spectrum of **1** (Table 1) indicated the presence of four methyls (*δ*_H_ 1.37 (*s*), 1.23 (*d*, *J* = 6.9), 1.04 (*d*, *J* = 8.0), 1.19 (*d*, *J* = 9.0) and one monosubstituted benzene ring [*δ*_H_ 7.76 (2H, *m*), 7.41 (2H, *m*) and 7.41 (1H, *m*)]. The ^13^C NMR data identified 38 carbon signals, categorized as 10 quaternary carbons (one ester, three olefinics, one orthoester and five oxygenated), 15 methines, 9 methylenes and 4 methyls (Table 1). The above data suggested that compound **1** and the previously identified molecule trigocherriolide B [8, 9] share a similar scaffold, except that one methylene signal at *δ*_H_ 5.17 (s), 5.07 (s) replaced the C-19 methine signal at *δ*_H_ 6.07 (s) in latter. Considering the molecular weight of **1** is 34 units less than that of trigocherriolide B, the compound should be 19-dechloro form of trigocherriolide B. The further HMBC cross peaks from H-3 to C-1 and C-19, H_2_-19 to C-2 and C-1, and H-1 to C-19 and C-2 located the *exo*-methylene at C-19. Thus, the gross structure of trigonolactone B (**1**) was established as shown (Fig. 2).

**Table 1:** ^1^H NMR and ^13^C NMR Data of **1**–**3**

No	**1**		**2**		**3**	
	*δ*_H_^a^	*δ*_C_^b^	*δ*_H_^a^	*δ*_C_^b^	*δ*_H_^c^	*δ*_C_^d^
1	6.30 (1H, s)	130.2 d	6.21 (1H, s)	130.2 d	6.22 (1H, s)	129.9 d
2		145.6 s		145.5 s		145.7 s
3	5.38 (1H, s)	80.0 d	5.35 (1H, s)	79.2 d	5.24 (1H, s)	80.3 d
4		83.5 s		83.2 s		83.3 s
5	3.87 (1H, s)	71.8 d	3.78 (1H, s)	71.2 d	3.92 (1H, s)	71.1 d
6		60.0 s		60.1 s		61.0 s
7	3.49 (1H, s)	62.0 d	3.01 (1H, s)	61.8 d	3.34 (1H, s)	62.9 d
8	4.79 (1H, s)	34.5 d	4.46 (1H, s)	34.2 d	4.48 (1H, s)	34.6 d
9		74.5 s		74.5 s		74.5 s
10		147.5 s		147.2 s		147.6 s
11	2.77 (1H, q, 6.9)	34.6 d	2.68 (1H, q, 6.9)	34.6 d	2.78 (1H, q, 6.9)	34.7 d
12	4.29 (1H, s)	79.7 d	4.29 (1H, s)	79.2 d	4.22 (1H, s)	79.5 d
13		72.1 s		72.4 s		73.2 s
14	4.39 (1H, s)	79.4 d	4.43 (1H, s)	79.2 d	4.67 (1H, s)	79.7 d
15		75.3 s		75.8 s		76.1 s
16α 16β	1.43 (1H, m) 1.71 (1H, m)	37.9 t	1.87 (1H, d, 18.0) 1.56 (1H, dd, 7.5, 18.0)	37.2 t	1.39 (1H, m) 1.65 (1H, m)	35.9 t
17α 17β	1.37 (3H, s)	23.8 q	4.78 (1H, d, 14.5) 4.54 (1H, d, 14.5)	67.8 t	3.90 (1H, d, 10.0) 3.61 (1H, d, 10.0)	65.2 t
18	1.23 (3H, d, 6.9)	13.9 q	1.23 (3H, d, 6.9)	13.9 q	1.20 (3H, d, 6.9)	13.7 q
19	5.17 (1H, s) 5.07 (1H, s)	111.2 t	5.11 (1H, s) 5.01 (1H, s)	111.3 t	5.13 (1H, s) 5.06 (1H, s)	110.0 t
20	3.91 (1H, d, 15.0) 3.75 (1H, d, 15.0)	62.4 t	3.56 (1H, br s) 3.42 (1H, br s)	62.8 t	3.74 (2H, m)	65.1 t
1′		179.7 s		179.2 s		178.5
2′	2.35 (1H, m)	42.8 d	2.31 (1H, m)	42.5 d	2.41 (1H, m)	42.0 d
3′α 3′β	1.69 (1H, m) 1.30 (1H, m)	35.1 t	1.67 (1H, m) 1.28 (1H, m)	34.9 t	1.67 (1H, m) 1.32 (1H, m)	34.9 t
4′α 4′β	1.30 (1H, m) 1.06 (1H, m)	31.6 t	1.30 (1H, m) 1.11 (1H, m)	31.3 t	1.29 (1H, m) 1.10 (1H, m)	31.1 t
5′	1.30 (2H, m)	27.2 t	1.30 (2H, m)	27.2 t	1.26 (2H, m)	26.8 t
6′	1.43 (2H, m)	28.3 t	1.38 (2H, m)	28.3	1.41 (2H, m)	28.6
7′α 7′β	1.48 (1H, m) 1.15 (1H, m)	27.8 t	1.52 (1H, m) 1.14 (1H, m)	28.2 t	1.47 (1H, m) 1.18 (1H, m)	27.6 t
8′α 8′β	1.21 (1H, m) 1.36 (1H, m)	37.5 t	1.40 (1H, m) 1.29 (1H, m)	37.9 t	1.19 (1H, m) 1.30 (1H, m)	38.2 t
9′	1.55 (1H, m)	24.9 d	1.77 (1H, m)	25.4 d	1.60 (1H, m)	25.1 d
10′	1.04 (3H, d, 8.0)	25.0 q	1.07 (3H, d, 7.5)	24.1 q	0.95 (3H, d, 6.5)	24.3 q
11′	1.19 (3H, d, 9.0)	18.8 q	1.18 (3H, d, 8.5)	18.8 q	1.16 (3H, d, 7.0)	18.6 q
1″		108.4 s		108.4 s		108.4 s
2″		138.5 s		138.4 s		138.4 s
3″/7″	7.76 (2H, m)	125.2 d	7.76 (1H, m)	125.2 d	7.71 (2H, m)	125.2 d
4″/6″	7.41 (2H, m)	128.2 d	7.43 (1H, m)	128.2 d	7.36 (2H, m)	128.0 d
5″	7.41 (1H, m)	129.5 d	7.41 (1H, m)	129.5 d	7.36 (1H, m)	129.4 d
1‴				170.1 s		
2‴				112.2 s		
3‴				161.8 s		
4‴			7.18 (1H, d, 10.0)	118.2 d		
5‴			7.49 (1H, t, 10.0)	135.9 d		
6‴			6.92 (1H, t, 10.0)	118.9 d		
7‴			7.70 (1H, d, 10.0)	129.4 d		
4-OH	3.67 (1H, s)					
13-OH	3.83 (1H, s)		3.94 (1H, s)		4.05 (1H, s)	
15-OH					3.40 (1H, s)	
3‴-OH			10.70 (1H, s)			

^a^Measured in CDCl_3_ at 400 MHz

^b^Measured in CDCl_3_ at 100 MHz

^c^Measured in CDCl_3_ at 500 MHz

^d^Measured in CDCl_3_ at 125 MHz

**Fig. 3:**
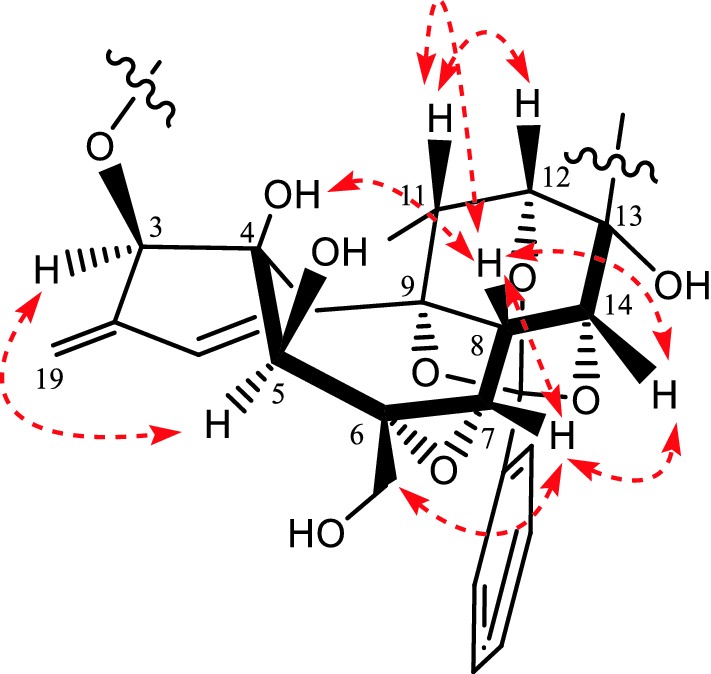
Key ROESY (  ) correlations of **1**

The relative configuration of **1** was elucidated by the ROESY experiment and compared with known compounds. The ROESY spectrum exhibited strong correlations of the axial H-11 to H-8 and H-12, orienting H-8 and H-12 on the *β*-orientation. The *β*-configurations of H-14, OH-4 and H-7 were established on the correlations of H-8 to H-7, H-14 and OH-4, meanwhile revealed that the 9, 12, 14-orthobenzoate was α-directed. The mutual ROESY correlations between H-3 and H-5 oriented the same *α*-configuration. Thus, the structure of **1** was elucidated as shown (Fig. 3).

The molecular formula of trigonolactone D (**2**) was indicated to be C_45_H_54_O_14_ by HRESIMS [*m*/*z* 841.3417 (M+Na)^+^, calcd 841.3411]. Compared the ^1^H and ^13^C NMR data (Table 1) with **1**, one more 1,2-disubstituted benzen ring (*δ*_H_ 7.18 (*d*, *J* = 10), 7.49 (*t*, *J* = 10), 6.92 (*t*, *J* = 10), 7.70 (*dd*, *J* = 1.5, 10) and ester carbonyl signal at *δ*_C_ 170.1 were easily assigned. The HMBC correlations of H-17α (*δ*_H_ 4.78) to C-13, C-15, C-16 and C-1′′′ confirmed compound **2** was the 17-(2-hydroxybenzoate) derivative of **1**. The relative configuration of **2** were elucidated by ROESY spectroscopy and similar with **1**.

The molecular formula of trigonolactone E (**3**) was established as C_38_H_50_O_12_ by HRESIME [*m*/*z* 721.3194 (M+Na)^+^, calcd 721.3200], which displayed one more oxygen atom than that of **1**. 1D and 2D NMR experiments confirmed that trigonolactone F (**3**) was the 17-hydroxymethy form of **1**. All other homonuclear (COSY and NOESY) and heteronuclear (HSQC and HMBC) correlations observed for **3** are like those of compound **1**, thus defining its planar structure and stereochemistry as depicted in Fig. 1.

Compounds **4**, **5** and **6** were identified by analysis of their spectroscopic data and comparison with literature data as trigocherriolides B and A, and trigocheriolide E [8–10], respectively.

### Anti-HIV-1 Activities

**Fig. 4:**
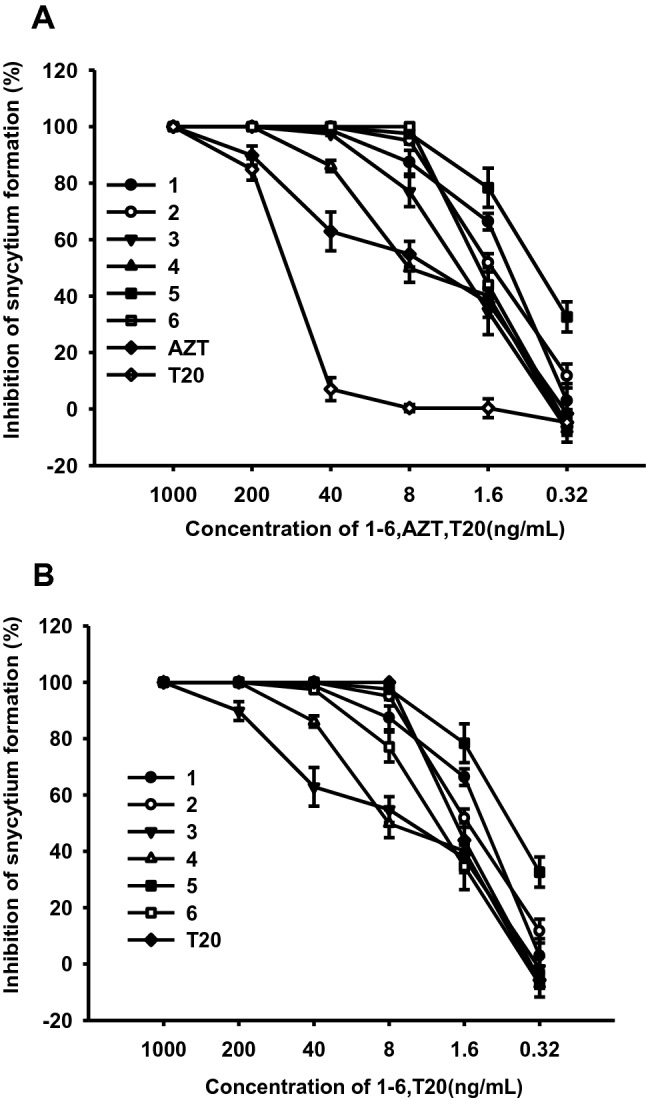
**a** The antiviral effects of **1**–**6** on HIV-1_IIIB_ in C8166 cells were assessed by syncytium formation; inhibitory activities of FuCS-1 on blocking fusion. **b** Inhibitory effect on cell-to-cell fusion between normal C8166 cells and HIV-1_IIIB_ infected H9 cells were measured by counting the syncytia formation. Data are expressed as means ± SD

Compounds **1**–**6** possessed low cytotoxicity to C8166 with CC_50_ were 16.47, 8.49, 17.47, 14.89, 15.52, 18.29 μg/mL and the compounds blocked 50% of the syncytia formation induced by HIV-1_IIIB_ at 1.06, 1.90, 0.59, 8.22, 2.87 and 1.50 ng/mL (Fig. 4a) (for positive controls, EC_50_ of AZT and T20 were 5.08 and 97.25 ng/mL).

**Fig. 5:**
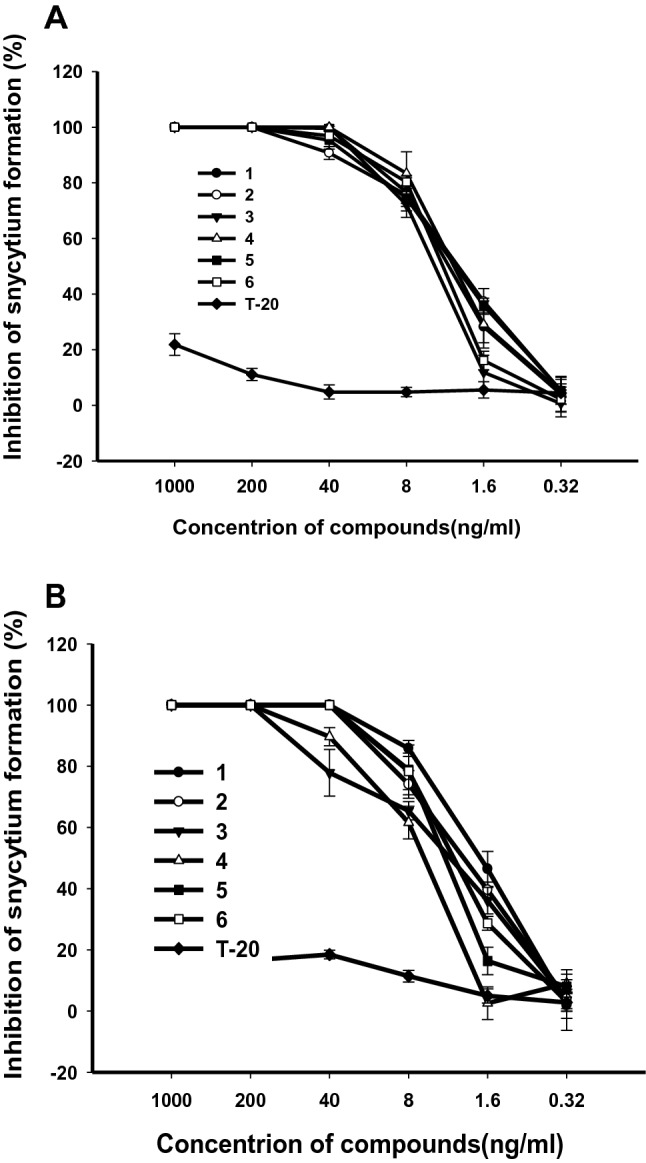
**a** The antiviral effects of **1**–**6** on pNL4-3gp41(36G)V38E,N42S in C8166 cells were assessed by syncytium formation. **b** The antiviral effects of **1**–**6** on pNL4-3gp41(36G)V38A,N42T in C8166 cells were assessed by syncytium formation. Data are expressed as means ± SD

T20 is the only FDA-approved first-generation HIV fusion inhibitor, which is being used for treatment of HIV/AIDS patients who have failed to respond to current antiretroviral drugs. Unfortunately, many patients are now failing to respond to enfuvirtide because it rapidly induces drug resistance in vitro and in vivo [11–13]. Thus, the inhibition assay of microtiter syncytium formation of the two T20-resistant HIV-1 strains, pNL4-3gp41(36G)V38E,N42S and pNL4-3gp41(36G)V38A,N42T in C8166 cells, were used to evaluate anti-HIV activity, respectively. All of the compounds showed significant inhibitoies with EC_50_s of 3.30, 2.72, 4.43, 2.97, 2.88 and 3.74 ng/mL for the former (Fig. 5a), and EC_50_s of 2.60, 5.83, 3.19,1.85, 3.43 and 3.81 ng/mL for the later (Fig. 5b) (EC_50_ of T20 > 1000 ng/mL).

### Mechanisms of Action

To address the action mechanisms, further experiments were carried out. HIV reverse transcriptase (RT) plays a very important role in the HIV replication, so the anti-HIV-1 RT activities of **1**–**6** were evaluated. The results demonstrated that all of them can slightly inhibited the enzymatic activity of purified recombinant HIV-1 RT with the EC_50_s of  > 300, 49.3,  > 300, 199.3, 267.4 and 191.4 μg/mL, which implied the compounds were not HIV RT inhibitor. In co-cultivation assay, compounds **1**–**6** effectively inhibited the fusion of H9/HIV-1_IIIB_ cells with uninfected C8166 cells, with EC_50_ values of 2.25, 4.62, 1.85, 8.73, 1.06 and 1.76 ng/mL (as the positive control, EC_50_ of T20 was 9.77 ng/mL), respectively (Fig. 4b), and when concentrations were 40 ng/mL, they inhibited about 90% of syncytia formation.

To further address action mechanisms of **1**–**6**, more experiments were carried out. In co-cultivation assay, those compounds effectively inhibited the fusion of H9/HIV-1_IIIB_ cells with uninfected C8166 cells but in RT study, it was found that they can’t inhibit HIV-1 RT activity at concentration as high as 300 μg/mL. These data supported that those compounds interfered HIV entry target cells possible.

Trigonothyrins A, B, C, D, F and G were tested for inhibitory activity against HIV-1 using the same method mentioned above [14, 15]. Only trigonothyrins C and F showed modest anti-HIV-1 activitise with EC_50_s of 2.19 and 0.13 μg/mL, and TI value of > 91.3 and 75.1 [14]. Analysis of those structures implied the macrolactone moiety and conjugative A ring appeared to be contributed for much higher antiviral activity. Compounds 19-Chlorine substituted **4**, **5** and **6** was less active than **1**, **2** and **3** respectively, indicating that the substitution pattern is not good for the activity. When 2-hydroxyl group were introduced at C-17, the bioactivity increased significantly, indicating the substitution at C-17 contribute to the improvement of inhibitory activity.

We found compounds **1**–**6** possesses low cytotoxicities to C8166 and exhibited potent anti-HIV activities. It not only inhibited replication of HIV-1 laboratory strains (HIV-1_IIIB_), but also inhibited T20-resistant strains (pNL4-3_gp41(36G)V38E,N42S_ and pNL4-3_gp41(36G)V38A,N42T_) that those compounds showed strong inhibitory activities against all T20-resistant strains with EC_50_ values about 10 ng/mL. It was interesting that compounds **1**–**6** were effective in these T20-resistant strains and the EC_50_s were similar in HIV-1IIIB. This result implied that FuSC-1 inhibit HIV with the mechanism different to HIV fusion inhibitor T20 and suggest that this class of compound can be further developed as an alternative entry inhibitor for treatment of patients with HIV-1/AIDS, those infected with T20-resistant variants.

## Experimental Section

### General Experimental Procedures

Optical rotation was carried out on a Perkin-Elmer model 241 polarimeter. IR spectra were measured in a Bio-Rad FTS-135 spectrometer with KBr pellets, whereas UV data were measured using a UV-210A spectrometer. Electrospary ionization-mass spectrometry (ESI-MS) and high-resolution (HR) ESI-MS were recorded with an APIQSTAR Pulsar 1 spectrometer (Advanced Biomics, Los Angeles). The 1D and 2D NMR spectra, including COSY, ROESY, HMBC, and HSQC experiments, were acquired at room temperature using a Bruker AM-400 and DRX-500 spectrometers operating at 400 and 500 MHz (^1^H) and 100 and 125 MHz (^13^C), respectively, with tetramethylsilane (TMS) as an internal standard. Multiplicities were determined using the DEPT pulse sequence. Column chromatography was performed on Si gel H (10–40 μm; Qingdao Marine Chemical Factory) and Sephadex LH-20 (40–70 μm, Amersham Pharmacia Biotech AB, Uppsala, Sweden). Semi-preparative HPLC was performed on a Zorbax SB-C18 (10 μm, Agilent Co. Ltd. Wilmington, DE) column (i.d. 9.4 × 250 mm), developed with CH_3_OH − H_2_O (60:40–50:50, 30 min) (flow rate, 2.0 mL/min; detection, UV 254 nm) at 35 °C. AZT, dextran sulfate (DS), T-20 were purchased from Sigma. Human polyclonal anti-HIV-1 serum was kindly donated by Dr. Hiroo Hoshino (Gunma University School of Medicine, Japan). Cell lines (C8166 and H9/HIV-1_IIIB_) were maintained in RPMI-1640 supplemented with 10% heat-inactivated newborn calf serum (Gibco). The HIV-1_IIIB_ virus was obtained from MRC, AIDS Reagent Project, UK. The 50% HIV-1 tissue culture infectious dose (TCID50) was determined and calculated by the Reed and Muench method. Virus stocks were stored in aliquots at − 70 °C [16].

### Extraction and Isolation

The leaves and twigs of *Trigonostemon lii* Y. T. Chang were collected in Xishuangbanna, Yunnan Province, People’s Republic of China, in November, 2008, and the plant sample was identified by Prof. Shun-Cheng Zhang of Xishuangbanna Institute of Botany, Chinese Academy of Sciences (CAS). A voucher specimen (KIB 08,110,211) was deposited at the State Key Laboratory of Phytochemistry and Plant Resources in West China, Kunming Institute of Botany, Chinese Academy of Science (CAS).

Air-dried, powdered leaves and twigs (50.0 kg) of *Trigonostemon lii* Y. T. Chang were extracted three times with Acetone at 50 °C. After removal of the solvent by evaporation, the residue was suspended in H_2_O and partitioned with petroleum ether. The petroleum ether (500 g) fraction was subjected to silica gel column chromatography with a gradient elution system of petroleum ether/acetone (100:0–30:70) to obtain eight fractions (A–H). Fraction F (80.0 g) was separated and purified by MPLC (MeOH–H_2_O, 85:15) to yield six fractions (C1–C6). Subfraction C3 was subjected to Sephadex LH-20 column chromatography (MeOH–H_2_O, 10:1), and then further purifying by the semi-preparative HPLC to yield compounds **1** (60.0 mg), **2** (20.0 mg), **3** (35.0 mg), **4** (100.0 mg), **5** (25.0 mg) and **6** (30.0 mg). All compounds were pure and dissolved in DMSO.

Trigonolactone B (**1**): white powder; [α]_D_^27^ = − 105.4, (*c* 0.14 CHCl_3_); UV (CHCl_3_) *λ*_max_ (log ε) 246 (4.18) nm; IR (KBr) *v*_max_ 3442, 2924, 1710, 1639 and 1453/cm; positive ESI-MS: *m/z* (100): 705 (100) [M+Na]^+^; HRESIMS: *m*/*z*: 705.3248 [M+Na]^+^, C_38_H_50_O_11_Na (calcd 705.3250).

Trigonolactone D (**2**): white powder; [α]_D_^27^ = − 120.0, (*c* 0.63 CHCl_3_); UV (CHCl_3_) *λ*_max_ (log ε) 244 (4.45) nm; IR (KBr) *v*_max_ 3443, 2925, 1704, 1678,1614, 1485 and 1461/cm; positive ESI-MS: *m/z* (100): 819 (18) [M+H]^+^, 841 (100) [M+Na]^+^; HRESIMS: *m*/*z*: 841.3417 [M+Na]^+^, C_45_H_54_O_14_ (calcd 841.3411).

Trigonolactone E (**3**): white powder; [α]_D_^25.1^ = − 107.6 (*c* 0.18, MeOH); V (MeOH) *λ*_max_ (log ε) 206 (4.03), 245 (4.20) nm; IR (KBr) *ν*_max_ 3441, 2932, 1710, 1641, 1453, 1331, 1269, 1084, 1030, 888, 752, 697/cm; positive ESI-MS: *m/z* (100): 721 (100) [M+Na]^+^; HRESIMS: *m/z*: 721.3194 [M+Na]^+^, C_38_H_50_O_12_Na (calcd 721.3200).

Trigocherriolide B (**4**): white powder; [α]_D_^27^ = − 122.5, (*c* 0.20 CHCl_3_); UV (CHCl_3_) *λ*_max_ (log ε) 257 (4.24) nm; IR (KBr) *v*_max_ 3455, 2931, 1713 and 1618/cm; positive ESI-MS: *m/z* (%): 717 (100) [M+H]^+^ 719 (35) [M+2]^+^; HRESIMS: *m*/*z*: 739.2846 [M+Na]^+^; C_38_H_49_O_11_NaCl (calcd 739.2861).

Trigocherriolide A (**5**): white powder; [α]_D_^27^ = − 126.4, (*c* 0.67 CHCl_3_); UV (CHCl_3_) *λ*_max_ (log ε) 248 (4.31) nm; IR (KBr) *v*_max_ 3444, 2925, 1710, 1677, 1640, 1615, 1485 and 1461/cm; positive ESI-MS: *m/z* (100): 853 (20) [M+H]^+^, 855 (8) [M+2+H]^+^, 875 (100) [M+Na]^+^, 875 (38) [M+Na]^+^; HRESIMS: *m*/*z*: 875.3029 [M+Na]^+^, C_45_H_53_O_14_NaCl (calcd 875.3021).

Trigocheriolide E (**6**): white powder; [α]_D_^24.7^ = − 112.8 (*c* 0.16, MeOH); UV (MeOH) *λ*_max_ (log ε) 257 (4.15) nm; IR (KBr) *ν*_max_ 3441, 2932, 1712, 1640, 1453, 1333, 1266, 1084, 1029, 812, 753, 697/cm; positive ESI-MS: *m/z* (100): 755 (100) [M+Na]^+^, 757 (42) [M+2+Na]^+^; HRESIMS: *m/z*: 755.2820 [M+Na]^+^, C_38_H_49_O_12_NaCl (calcd 755.2810).

### MTT-Based Cytotoxicity Assay

Cellular toxicity of compounds was assessed by MTT method. Briefly, cells were seeded on a microtiter plate in the absence or presence of various concentrations of compounds in triplicate and incubated at 37 °C in a humid atmosphere of 5% CO_2_ for 3 days. Twenty microliters of MTT reagent (5 mg/mL in PBS) was added to each well, then incubated at 37 °C for 4 h, 100 μL of 50% DMF-20% SDS was added. After the formazan was dissolved completely, the plates were read on a Bio-Tek ELx 800 ELISA reader at 595 nm/630 nm (A595/630). The cytotoxic concentration that caused the reduction of viable cells by 50% (CC_50_) was calculated from dose–response curve [17].

### Syncytia Assay

In the presence of 100 μL various concentrations of compounds, C8166 cells (4 × 10^5^/mL) were infected with virus (HIV-1_IIIB_) at a multiplicity of infection (M.O.I) of 0.06. The final volume per well was 200 μL. Control assays were performed without the testing compounds in HIV-1_IIIB_ infected and uninfected cultures. AZT was included as positive control. After 3 days of culture, the cytopathic effect (CPE) was measured by counting the number of syncytia (multinucleated giant cell). Percentage inhibition of syncytia formation was calculated and 50% effective concentration (EC_50_) was calculated [18, 19].

### Co-cultivation Assay

C8166 cells (3 × 10^4^) co-cultured with 1 × 10^4^ virus (HIV-1_IIIB_) infected H9 cells in the presence or absence of the compound with various concentrations at 37 °C in a humidified atmosphere of 5% CO_2_. T20 was used as positive control. After 6 h incubation, the number of syncytia was scored under an inverted microscope [18].

### RT (Reverse Transcriptase) Assay

HIV-1 RT activity was measured by ELISA RT kit (Roche) using a commercially available kit according to the protocol provided by the manufacturer. Samples were incubated with DIG-labeled-reaction mixture at 37 °C for 15 h. Anti-DIG-POD solution was added afterward followed by substrate ABTS. The absorbance at 405/490 nm (A405/490) was determined in the ELISA reader [20].

## Electronic supplementary material

Below is the link to the electronic supplementary material.

Supplementary file1 Supplementary data (^1^H and^13^C NMR data of **1**-**6**; Physical data of **1**-**6**; 1D and 2D NMR of **1**-**3**; Anti-HIV data of **1**-**6**; detailed experimental procedures) can be found. (DOC 16150 kb)
