# Supplementary material for: Daphnane Diterpenoids from Trigonostemon lii and Inhibition Activities Against HIV-1
Source: Nat Prod Bioprospect. 2020 Feb 11;10(1):37–44. doi: 10.1007/s13659-020-00231-7 (PMC7046902; doi:10.1007/s13659-020-00231-7)

**Daphnane diterpenoids from *Trigonostemon lii* and inhibition activities against HIV-1**

Cheng-Jian Tan^a,c, #^, Shi-Fei Li^a,d #^, Ning Huang^b,e, #^, Yu Zhang^a^, Ying-Tong Di^a^, Yong-Tang Zheng^b,*^ Xiao-Jiang Hao^a^,*

^a^ *State Key Laboratory of Phytochemistry and Plant Resources in West China, Kunming Institute of Botany, Chinese Academy of Sciences, Kunming 650204, Yunnan, P. R. China*

^b^ *Key Laboratory of Bioactive Peptides of Yunnan Province/Key Laboratory of Animal Models and Human Disease Mechanisms of the Chinese Academy of Sciences, Center for Biosafety Mega-Science, Kunming Institute of Zoology, Chinese Academy of Sciences, Kunming 650223, Yunnan, P. R. China*

^c^ School of ethnic medicine, Guizhou Minzu University, Guiyang 550025, Guizhou, *P. R. China*

^d^ Institute of Molecular Science, Shanxi University, Taiyuan 030006, Shanxi, *P. R.* China

^e^ *School of Basic Medical, Kunming Medical University, Kunming 650500, Yunnan, P. R.China*

* Corresponding author. Fax: (+)(86) 871-5223070. Tel: (86) 871-5223263.

E-mail address: [*haoxj@mail.kib.ac.cn*](mailto:haoxj@mail.kib.ac.cn); [*zhengyt@mail.kiz.ac.cn*](mailto:zhengyt@mail.kiz.ac.cn)

^#^These authors contributed equally to this work

**Contents**

**S1.** Experimental section.

**S2.** ^1^H-NMR (Table 1) and ^13^C-NMR (Table 2) data of **1**-**6**.

**S3.** Physical data of **1**-**6**.

**S4.** Anti-HIV data of **1**-**6**.

**Figure S5.** ESI mass spectrum of **1**.

**Figure S6.** HRESI mass spectrum of **1**.

**Figure S7.** ^1^H NMR (400 MHz) spectrum of **1** in CDCl_3_.

**Figure S8**. ^13^C NMR (100 MHz) spectrum of **1** in CDCl_3_.

**Figure S9.** HSQC spectrum of **1** in CDCl_3_.

**Figure S10.** ^1^H-^1^H COSY spectrum of **1** in CDCl_3_.

**Figure S11.** HMBC spectrum of **1** in CDCl_3_.

**Figure S12.** ROESY spectrum of **1** in CDCl_3_.

**Figure S13.** ESI mass spectrum of **2**.

**Figure S14.** HRESI mass spectrum of **2**.

**Figure S15.** ^1^H NMR (400 MHz) spectrum of **2** in CDCl_3_.

**Figure S16**. ^13^C NMR (100 MHz) spectrum of **2** in CDCl_3_.

**Figure S17.** HSQC spectrum of **2** in CDCl_3_.

**Figure S18.** ^1^H-^1^H COSY spectrum of **2** in CDCl_3_.

**Figure S19.** HMBC spectrum of **2** in CDCl_3_.

**Figure S20.** ROESY spectrum of **2** in CDCl_3_.

**Figure S21.** ESI mass spectrum of **3**.

**Figure S22.** HRESI mass spectrum of **3**.

**Figure S23.** ^1^H NMR (500 MHz) spectrum of **3** in CDCl_3_.

**Figure S24**. ^13^C NMR (125 MHz) spectrum of **3** in CDCl_3_.

**Figure S25.** HSQC spectrum of **3** in CDCl_3_.

**Figure S26.** ^1^H-^1^H COSY spectrum of **3** in CDCl_3_.

**Figure S27.** HMBC spectrum of **3** in CDCl_3_.

**Figure S28.** ROESY spectrum of **3** in CDCl_3_.

**S1. Experimental section**

***A. General Experimental Procedures***

Optical rotation was carried out on a Perkin-Elmer model 241 polarimeter. IR spectra were measured in a Bio-Rad FTS-135 spectrometer with KBr pellets, whereas UV data were measured using a UV-210A spectrometer. Electrospary ionization-mass spectrometry (ESI-MS) and high-resolution (HR) ESI-MS were recorded with an APIQSTAR Pulsar 1 spectrometer (Advanced Biomics, Los Angeles). The 1D and 2D NMR spectra, including COSY, ROESY, HMBC, and HSQC experiments, were acquired at room temperature using a Bruker AM-400 and DRX-500 spectrometers operating at 400 and 500 MHz (^1^H) and 100 and 125 MHz (^13^C), respectively, with tetramethylsilane (TMS) as an internal standard. Multiplicities were determined using the DEPT pulse sequence. Column chromatography was performed on Si gel H (10-40 *μ*m; Qingdao Marine Chemical Factory) and Sephadex LH-20 (40-70 *μ*m, Amersham Pharmacia Biotech AB, Uppsala, Sweden). Semi-preparative HPLC was performed on a Zorbax SB-C18 (10 *μ*m, Agilent Co. Ltd. Wilmington, DE) column (i.d. 9.4 × 250 mm), developed with CH_3_OH−H_2_O (60:40-50:50, 30 min) (flow rate, 2.0 mL/min; detection, UV 254 nm) at 35 ºC.

***B. Plant Material***

The leaves and twigs of *Trigonostemon lii* Y. T. Chang were collected in Xishuangbanna, Yunnan Province, People’s Republic of China, in November, 2008, and the plant sample was identified by Prof. Shun-Cheng Zhang of Xishuangbanna Institute of Botany, Chinese Academy of Sciences (CAS). A voucher specimen (KIB 08110211) was deposited at the State Key Laboratory of Phytochemistry and Plant Resources in West China, Kunming Institute of Botany, Chinese Academy of Science (CAS).

***C. Extraction and Isolation***

Air-dried, powdered leaves and twigs (50.0 kg) of *Trigonostemon lii* Y. T. Chang were extracted three times with Acetone at 50°C. After removal of the solvent by evaporation, the residue was suspended in H_2_O and partitioned with petroleum ether. The petroleum ether (500 g) fraction was subjected to silica gel column chromatography with a gradient elution system of petroleum ether/acetone (100:0–30:70) to obtain eight fractions (A–H). Fraction F (80.0 g) was separated and purified by MPLC (MeOH–H_2_O, 85:15) to yield six fractions (C1–C6). Subfraction C3 was subjected to Sephadex LH-20 column chromatography (MeOH–H_2_O, 10:1), and then further purifying by the semi-preparative HPLC to yield compounds **1** (60.0 mg), **2** (20.0 mg), **3** (35.0 mg), **4** (100.0 mg), **5** (25.0 mg), and **6** (30.0 mg).

***D. Anti-HIV activity assay***

**a. *Chemicals*** AZT and T20 were purchased from Sigma. ***Cells and virus*** Cell lines (C8166 and H9/HIV-1_IIIB_) were maintained in RPMI-1640 supplemented with 10% heat-inactivated newborn calf serum (Gibco). The HIV-1_IIIB_ viruse was obtained from MRC, AIDS Reagent Project, UK. The 50% HIV-1 tissue culture infectious dose (TCID50) was determined and calculated by the Reed and Muench method. Virus stocks were stored in aliquots at -70 °C ^[1]^.

**b. *MTT-based cytotoxicity assay.*** Cellular toxicity of compounds was assessed by MTT method. Briefly, cells were seeded on a microtiter plate in the absence or presence of various concentrations of compounds in triplicate and incubated at 37 °C in a humid atmosphere of 5% CO_2_ for 3 days. Twenty microliters of MTT reagent (5 mg/mL in PBS) was added to each well, then incubated at 37 °C for 4h, 100 μL of 50% DMF-20% SDS was added. After the formazan was dissolved completely, the plates were read on a Bio-Tek ELx 800 ELISA reader at 595nm/630 nm (A595/630). The cytotoxic concentration that caused the reduction of viable cells by 50% (CC_50_) was calculated from dose-response curve ^[2]^.

**c. *Syncytia assay.*** In the presence of 100 μL various concentrations of compounds, C8166 cells (4×10^5^/mL) were infected with virus (HIV-1_IIIB_) at a multiplicity of infection (M.O.I) of 0.06. The final volume per well was 200 μL. Control assays were performed without the testing compounds in HIV-1_IIIB_ infected and uninfected cultures. AZT was included as positive control. After 3 days of culture, the cytopathic effect (CPE) was measured by counting the number of syncytia (multinucleated giant cell). Percentage inhibition of syncytia formation was calculated and 50% effective concentration (EC_50_) was calculated ^[3]^.

**d*. Co-cultivation assay.*** C8166 cells (3×10^4^) co-cultured with 1×10^4^ virus (HIV-1_IIIB_) infected H9 cells in the presence or absence of the compound with various concentrations at 37 °C in a humidified atmosphere of 5% CO_2_. Dextran sulfate (DS) was used as positive control. After 6 h incubation, the number of syncytia was scored under an inverted microscope ^[3]^.

**e. *RT (reverse transcriptase) assay.*** HIV-1 RT activity was measured by ELISA RT kit (Roche) using a commercially available kit according to the protocol provided by the manufacturer. Samples were incubated with DIG-labeled-reaction mixture at 37 °C for 15 h. Anti-DIG-POD solution was added afterward followed by substrate ABTS. The absorbance at 405/490 nm (A405/490) was determined in the ELISA reader ^[4]^.

**References**

[1] A.M. Vandamme, M. Witvrou, C. Pannecouque, J. Balzarini, K. Van Laethem, J.C. Schmit, J. Desmyter, E. De Clercq, Evaluating clinical isolates for their phenotypic and genotypic resistance against anti-HIV drugs, in: D. Kinchington, R.F. Schinazi (Eds.), Antiviral Methods and protocols, Humanae Press, Clifton, 2000, pp. 223-231.

[2] Y. T. Zheng, W. F. Zhang, K. L. Ben, J. H. Wang, *Immunopharmacol. mmunotoxicol*. **1995**, *17*, 69-79.

[3] Y. T. Zheng, K. L. Ben, S. W. Jin, *Acta Pharmacol. Sin*. **2000**, *21*, 179-182.

[4] Q. Wang, Z. H. Ding, J. K. Liu, Y. T. Zheng, *Antiviral Res*. **2004**, *64*,189-194.

**S2.** *Structural elucidation compounds* ***1****-****6***

**Table 1**. ^1^H NMR Data of **1**-**6**

| NO. | **1** | **2** | **3** | **4** | **5** | **6** |
| --- | --- | --- | --- | --- | --- | --- |
| 1 | 6.30 (1H, s) | 6.21 (1H, s) | 6.22 (1H, s) | 6.48 (1H, s ) | 6.42 (1H, s) | 6.36 (1H, s ) |
| 3 | 5.38 (1H, s) | 5.35 (1H, s) | 5.24 (1H, s) | 5.38 (1H, s ) | 5.32 (1H, s) | 5.22 (1H, s ) |
| 5 | 3.87 (1H, s) | 3.78 (1H, s) | 3.92 (1H, s) | 3.93 (1H, s ) | 3.78 (1H, s) | 3.91 (1H, s ) |
| 7 | 3.49 (1H, s) | 3.01 (1H, s) | 3.34 (1H, s) | 3.41 (1H, s) | 2.99 (1H, s) | 3.24 (1H, s ) |
| 8 | 4.79 (1H, s) | 4.46 (1H, s) | 4.48 (1H, s) | 4.74 (1H, s) | 4.45 (1H, s) | 4.41 (1H, s ) |
| 11 | 2.77 (1H, q, 6.9) | 2.68 (1H, q, 6.9) | 2.78 (1H, q, 6.9) | 2.74 (1H, q, 6.9) | 2.71 (1H, q, 6.9) | 2.78 (1H,q, 6.9) |
| 12 | 4.29 (1H, s) | 4.29 (1H, s) | 4.22 (1H, s ) | 4.29 (1H, s) | 4.31 (1H, s) | 4.21 (1H, s ) |
| 14 | 4.39 (1H, s) | 4.43 (1H, s) | 4.67 (1H, s ) | 4.39 (1H, s) | 4.48 (1H, s) | 4.59 (1H, s ) |
| 16α  16β | 1.43 (1H, m)  1.71 (1H, m) | 1.87 (1H, d, 18.0)  1.56 (1H, dd, 7.5, 18.0) | 1.39 (1H, m)  1.65 (1H, m) | 1.47 (1H, m)  1.73 (1H, m) | 1.84 (1H, d, 18.0)  1.57 (1H, dd, 7.5, 18.0) | 1.40 (1H, m)  1.62 (1H, m) |
| 17α  17β | 1.37 (3H, s) | 4.78 (1H, d, 14.5)  4.54 (1H, d, 14.5) | 3.90 (1H, d, 10.0)  3.61 (1H, d, 10.0) | 1.39 (3H, s) | 4.80 (1H, d, 15.0)  4.57 (1H, d, 15.0) | 3.88 (1H, d, 12.0)  3.59 (1H, d, 12.0) |
| 18 | 1.23 (3H, d, 6.9) | 1.23 (3H, d, 6.9) | 1.20 (3H, d, 6.9) | 1.23 (3H, d, 6.9) | 1.24 (3H, d, 6.9) | 1.19 (3H, d, 6.9) |
| 19 | 5.17 (1H, s)  5.07 (1H, s) | 5.11 (1H, s)  5.01 (1H, s) | 5.13 (1H, s)  5.06 (1H, s) | 6.07 ( 1H, s ) | 6.00 (1H, s) | 6.04 ( 1H, s ) |
| 20 | 3.91 (1H, d, 15.0)  3.75 (1H, d, 15.0) | 3.56 (1H, br s)  3.42 (1H, br s) | 3.74 (2H, m) | 3.83 (2H, br s ) | 3.59 (1H,d, 15.5)  3.48 (1H,d, 15.5) | 3.82 (1H, d, 10.9)  3.58 (1H, d, 10.9) |
| 2′ | 2.35 (1H, m) | 2.31 (1H, m) | 2.41 (1H, m) | 2.41 (1H, m) | 2.31 (1H, m) | 2.41 (1H, m) |
| 3′α  3′β | 1.69 (1H, m)  1.30 (1H, m) | 1.67 (1H, m)  1.28 (1H, m) | 1.67 (1H, m)  1.32 (1H, m) | 1.70 (1H, m)  1.36 (1H, m) | 1.67 (1H, m)  1.30 (1H, m) | 1.64 (1H, m)  1.31 (1H, m) |
| 4′α  4′β | 1.30 (1H, m)  1.06 (1H, m) | 1.30 (1H, m)  1.11 (1H, m) | 1.29 (1H, m)  1.10 (1H, m) | 1.35 (1H, m)  1.09 (1H, m) | 1.31 (1H, m)  1.12 (1H, m) | 1.28 (1H, m)  1.08 (1H, m) |
| 5′ | 1.30 (2H, m ) | 1.30 (2H, m) | 1.26 (2H, m) | 1.31 (2H, m ) | 1.31 (2H, m) | 1.23 (2H, m) |
| 6′ | 1.43 (2H, m ) | 1.38 (2H, m) | 1.41 (2H, m) | 1.41 (2H, m ) | 1.38 (2H, m) | 1.38 (1H, m)  1.11 (1H, m) |
| 7′α  7′β | 1.48 (1H, m )  1.15 (1H, m ) | 1.52 (1H, m)  1.14 (1H, m) | 1.47 (1H, m)  1.18 (1H, m) | 1.48 (1H, m )  1.18 (1H, m ) | 1.46 (1H, m)  1.16 (1H, m) | 1.42 (1H, m)  1.17 (1H, m) |
| 8′α  8′β | 1.21 (1H, m)  1.36 (1H, m) | 1.40 (1H, m)  1.29 (1H, m) | 1.19 (1H, m)  1.30 (1H, m) | 1.23 (1H, m)  1.40 (1H, m) | 1.40 (1H, m)  1.30 (1H, m) | 1.19 (1H, m)  1.29 (1H, m) |
| 9′ | 1.55 (1H, m) | 1.77 (1H, m) | 1.60 (1H, m) | 1.59 (1H, m) | 1.76 (1H, m) | 1.60 (1H, m) |
| 10′ | 1.04 (3H, d, 8.0) | 1.07 (3H, d, 7.5 ) | 0.95 (3H, d, 6.5) | 1.04 (3H, d, 8.0) | 1.09 (3H, d, 8.0 ) | 0.94 (3H, d, 6.4) |
| 11′ | 1.19 (3H, d, 9.0 ) | 1.18 (3H, d, 8.5 ) | 1.16 (3H, d, 7.0) | 1.20 (3H, d, 9.0 ) | 1.17 (3H, d, 9.0 ) | 1.13 (3H, d, 7.0) |
| 3′′/7′′ | 7.76 (2H, m ) | 7.76 (1H, m ) | 7.71 (2H, m ) | 7.76 (2H, m ) | 7.76 (1H, m ) | 7.69 (2H, m ) |
| 4′′/6′′ | 7.41 (2H, m ) | 7.43 (1H, m ) | 7.36 (2H, m ) | 7.43 (2H, m ) | 7.43 (1H, m ) | 7.36 (2H, m ) |
| 5′′ | 7.41 (1H, m ) | 7.41 ( 1H, m ) | 7.36 (1H, m ) | 7.41 (1H, m ) | 7.41 ( 1H, m ) | 7.36 (1H, m ) |
| 4′′′ |  | 7.18 (1H, d, 10.0) |  |  | 7.18 (1H, d, 10.0) |  |
| 5′′′ |  | 7.49 (1H, t, 10.0) |  |  | 7.49 (1H, t, 10.0) |  |
| 6′′′ |  | 6.92 (1H, t, 10.0) |  |  | 6.92 (1H, t, 10.0) |  |
| 7′′′ |  | 7.70 (1H, d, 10.0) |  |  | 7.70 (1H, d, 10.0) |  |
| 4-OH | 3.67 (1H, s) |  |  | 3.70 (1H, s) |  |  |
| 13-OH | 3.83(1H, s) | 3.94 (1H, s ) | 4.05 (1H, s) | 3.86 (1H, s) | 3.94 (1H, s) | 4.03 (1H, s) |
| 15-OH |  |  | 3.40 (1H, s) | 2.80 (1H, s) |  | 3.28 (1H, s) |
| 3′′′-OH |  | 10.70 (1H, s) |  |  | 10.70 (1H, s) |  |

**Table 2. ^13^C NMR Data of 1-**6

| NO. | **1** | **2** | **3** | **4** | **5** | **6** |
| --- | --- | --- | --- | --- | --- | --- |
| 1 | 130.2 d | 130.2 d | 129.9 d | 125.7 d | 125.7 d | 125.4 d |
| 2 | 145.6 s | 145.5 s | 145.7 s | 139.7 s | 139.6 s | 139.8 s |
| 3 | 80.0 d | 79.2 d | 80.3 d | 78.6 d | 79.9 d | 78.8 d |
| 4 | 83.5 s | 83.2 s | 83.3 s | 83.8 s | 83.8 s | 83.5 s |
| 5 | 71.8 d | 71.2 d | 71.1 d | 71.6 d | 71.0 d | 70.8 d |
| 6 | 60.0 s | 60.1 s | 61.0 s | 60.5 s | 60.3 s | 61.2 s |
| 7 | 62.0 d | 61.8 d | 62.9 d | 62.3 d | 61.8 d | 62.9 d |
| 8 | 34.5 d | 34.2 d | 34.6 d | 34.6 d | 34.3 d | 34.6 d |
| 9 | 74.5 s | 74.5 s | 74.5 s | 74.6 s | 74.6 s | 74.6 s |
| 10 | 147.5 s | 147.2 s | 147.6 s | 149.2 s | 149.0 s | 149.4 s |
| 11 | 34.6 d | 34.6 d | 34.7 d | 34.7 d | 34.6 d | 34.5 d |
| 12 | 79.7 d | 79.2 d | 79.5 d | 79.6 d | 79.2 d | 79.4 d |
| 13 | 72.1 s | 72.4 s | 73.2 s | 72.1 s | 72.3 s | 73.1 s |
| 14 | 79.4 d | 79.2 d | 79.7 d | 79.2 d | 79.0 d | 79.7 d |
| 15 | 75.3 s | 75.8 s | 76.1 s | 75.5 s | 76.0 s | 76.2 s |
| 16 | 37.9 t | 37.2 t | 35.9 t | 38.3 t | 37.1 t | 35.9 t |
| 17 | 23.8 q | 67.8 t | 65.2 t | 23.7 q | 67.8 t | 65.2 t |
| 18 | 13.9 q | 13.9 q | 13.7 q | 13.9 q | 13.6 q | 13.7 q |
| 19 | 111.2 t | 111.3 t | 110.0 t | 114.5 d | 114.5 d | 114.3 d |
| 20 | 62.4 t | 62.8 t | 65.1 t | 63.6 t | 63.4 t | 65.3 t |
| 1′ | 179.7 s | 179.2 s | 178.5 | 178.7 s | 178.8 s | 178.0 s |
| 2′ | 42.8 d | 42.5 d | 42.0 d | 42.3 d | 42.5 d | 41.8 d |
| 3′ | 35.1 t | 34.9 t | 34.9 t | 34.9 t | 34.7 t | 34.8 t |
| 4′ | 31.6 t | 31.3 t | 31.1 t | 31.4 t | 31.0 t | 31.1 t |
| 5′ | 27.2 t | 27.2 t | 26.8 t | 27.0 t | 27.1 t | 26.8 t |
| 6′ | 28.3 t | 28.3 t | 28.6 t | 28.2 t | 28.4 t | 28.6 t |
| 7′ | 27.8 t | 28.2 t | 27.6 t | 27.8 t | 28.0 t | 27.1 t |
| 8′ | 37.5 t | 37.9 t | 38.2 t | 37.8 t | 37.9 t | 38.2 t |
| 9′ | 24.9 d | 25.4 d | 25.1 d | 25.1 d | 25.6 d | 25.3 d |
| 10′ | 25.0 q | 24.1 q | 24.3 q | 24.9 q | 24.2 q | 24.3 q |
| 11′ | 18.8 q | 18.8 q | 18.6 q | 18.6 q | 18.8 q | 18.6 q |
| 1′′ | 108.4 s | 108.4 s | 108.4 s | 108.4 s | 108.4 s | 108.4 s |
| 2′′ | 138.5 s | 138.4 s | 138.4 s | 138.4 s | 138.2 s | 138.3 s |
| 3′′/7′′ | 125.2 d | 125.2 d | 125.2 d | 125.2 d | 125.2 d | 125.2 d |
| 4′′/6′′ | 128.2 d | 128.2 d | 128.0 d | 128.2 d | 128.2 d | 128.2 d |
| 5′′ | 129.5 d | 129.5 d | 129.4 d | 129.5 d | 129.5 d | 129.5 d |
| 1′′′ |  | 170.1 s |  |  | 170.2 s |  |
| 2′′′ |  | 112.2 s |  |  | 112.1 s |  |
| 3′′′ |  | 161.8 s |  |  | 161.7 s |  |
| 4′′′ |  | 118.2 d |  |  | 118.2 d |  |
| 5′′′ |  | 135.9 d |  |  | 136.1 d |  |
| 6′′′ |  | 118.9 d |  |  | 119.0 d |  |
| 7′′′ |  | 129.4 d |  |  | 129.5 d |  |

Trigonolactone B **(1)**：white powder; [α]^27^_D_ = –105.4, (*c* 0.14 CHCl_3_ ); UV (CHCl_3_) *λ*_max_ (log ε) 246 (4.18) nm; IR (KBr) *v*_max_ 3442, 2924, 1710, 1639 and 1453 cm^–1^; positive ESI-MS: *m/z* (100): 705 (100) [M+Na]^+^; HRESIMS: *m*/*z*: 705.3248 [M+Na]^+^, C_38_H_50_O_11_Na (calcd 705.3250).

Trigonolactone D (**2**): white powder; [α]^27^_D_ = –120.0, (*c* 0.63 CHCl_3_ ); UV (CHCl_3_) *λ*_max_ (log ε) 244 (4.45) nm; IR (KBr) *v*_max_ 3443, 2925, 1704, 1678,1614, 1485 and 1461 cm^–1^; positive ESI-MS: *m/z* (100): 819 (18) [M+H]^+^, 841 (100) [M+Na]^+^; HRESIMS: *m*/*z*: 841.3417 [M+Na]^+^, C_45_H_54_O_14_ (calcd 841.3411).

Trigonolactone E **(3):** white powder; [α]^25.1^_D_ = –107.6 (*c* 0.18 , MeOH); V (MeOH) *λ*_max_ (log ε) 206 (4.03), 245 (4.20) nm; IR (KBr) *ν*_max_ 3441, 2932, 1710, 1641, 1453, 1331, 1269, 1084, 1030, 888, 752, 697 cm^–1^; positive ESI-MS: *m/z* (100): 721 (100) [M+Na]^+^; HRESIMS: *m/z*: 721.3194 [M+Na]^+^, C_38_H_50_O_12_Na (calcd 721.3200).

Trigocherriolide B (**4**)：white powder; [α]^27^_D_ = –122.5, (*c* 0.20 CHCl_3_ ); UV (CHCl_3_) *λ*_max_ (log ε) 257 (4.24) nm; IR (KBr) *v*_max_ 3455, 2931, 1713 and 1618 cm^–1^; positive ESI-MS: *m/z* (%): 717 (100) [M+H]^+^ 719 (35) [M+2]^+^; HRESIMS: *m*/*z*: 739.2846 [M+Na]^+^; C_38_H_49_O_11_NaCl (calcd 739.2861).

Trigocherriolide A (**5**): white powder; [α]^27^_D_ = –126.4, (*c* 0.67 CHCl_3_ ); UV (CHCl_3_) *λ*_max_ (log ε) 248 (4.31) nm; IR (KBr) *v*_max_ 3444, 2925, 1710, 1677, 1640, 1615, 1485 and 1461 cm^–1^; positive ESI-MS: *m/z* (100): 853 (20) [M+H]^+^, 855 (8) [M+2+H]^+^, 875 (100) [M+Na]^+^, 875 (38) [M+Na]^+^; HRESIMS: *m*/*z*: 875.3029 [M+Na]^+^, C_45_H_53_O_14_NaCl (calcd 875.3021).

Trigocheriolide E (**6**)**:** white powder; [α]^24.7^_D_ = –112.8 (*c* 0.16 , MeOH); UV (MeOH) *λ*_max_ (log ε) 257 (4.15) nm; IR (KBr) *ν*_max_ 3441, 2932, 1712, 1640, 1453, 1333, 1266, 1084, 1029, 812, 753, 697 cm^–1^; positive ESI-MS: *m/z* (100): 755 (100) [M+Na]^+^, 757 (42) [M+2+Na]^+^; HRESIMS: *m/z*: 755.2820 [M+Na]^+^, C_38_H_49_O_12_NaCl (calcd 755.2810).

**S4.** Anti-HIV data of **1**-**6**.

**Table 1**. Cytotoxicity and Anti-HIV-1 Activity of **1**-**6.**

| Compounds | cytotoxicity | anti-HIV-1 | selectivity index |
| --- | --- | --- | --- |
|  | CC_50_(μg/mL) | EC_50_(ng/mL) | CC_50_/EC_50_ |
| **1** | 16.47 | 1.06 | 15337.74 |
| **2** | 8.49 | 1.90 | 4468.42 |
| **3** | 17.47 | 0.59 | 29610.17 |
| **4** | 14.89 | 8.22 | 1811.43 |
| **5** | 15.52 | 2.87 | 5407.67 |
| **6** | 18.29 | 1.50 | 12193.33 |
| AZT(positive control) | 1006.69 | 5.08 | 198167.32 |

Fig. 1. (A) The antiviral effects of **1**-**6** on HIV-1_IIIB_ in C8166 cells were assessed by syncytium formation; inhibitory activities of FuCS-1 on blocking fusion. (B) Inhibitory effect on cell-to-cell fusion between normal C8166 cells and HIV-1_IIIB_ infected H9 cells were measured by counting the syncytia formation. Data are expressed as means ± SD.

Fig. 2. (A) The antiviral effects of **1**-**6** on pNL4-3gp41(36G)V38E,N42S in C8166 cells were assessed by syncytium formation. (B) The antiviral effects of **1**-**6** on pNL4-3gp41(36G)V38A,N42T in C8166 cells were assessed by syncytium formation. Data are expressed as means ± SD.

***Table 2. Inhibition of 1-6 on recombinant HIV-1 RT activity***

| Compounds | EC50 (μg/mL) |
| --- | --- |
| 1 | >300 |
| 2 | 49.3 |
| 3 | >300 |
| 4 | 199.3 |
| 5 | 267.4 |
| 6 | 191.4 |

**Figure S5.** ESI mass spectrum of **1**.


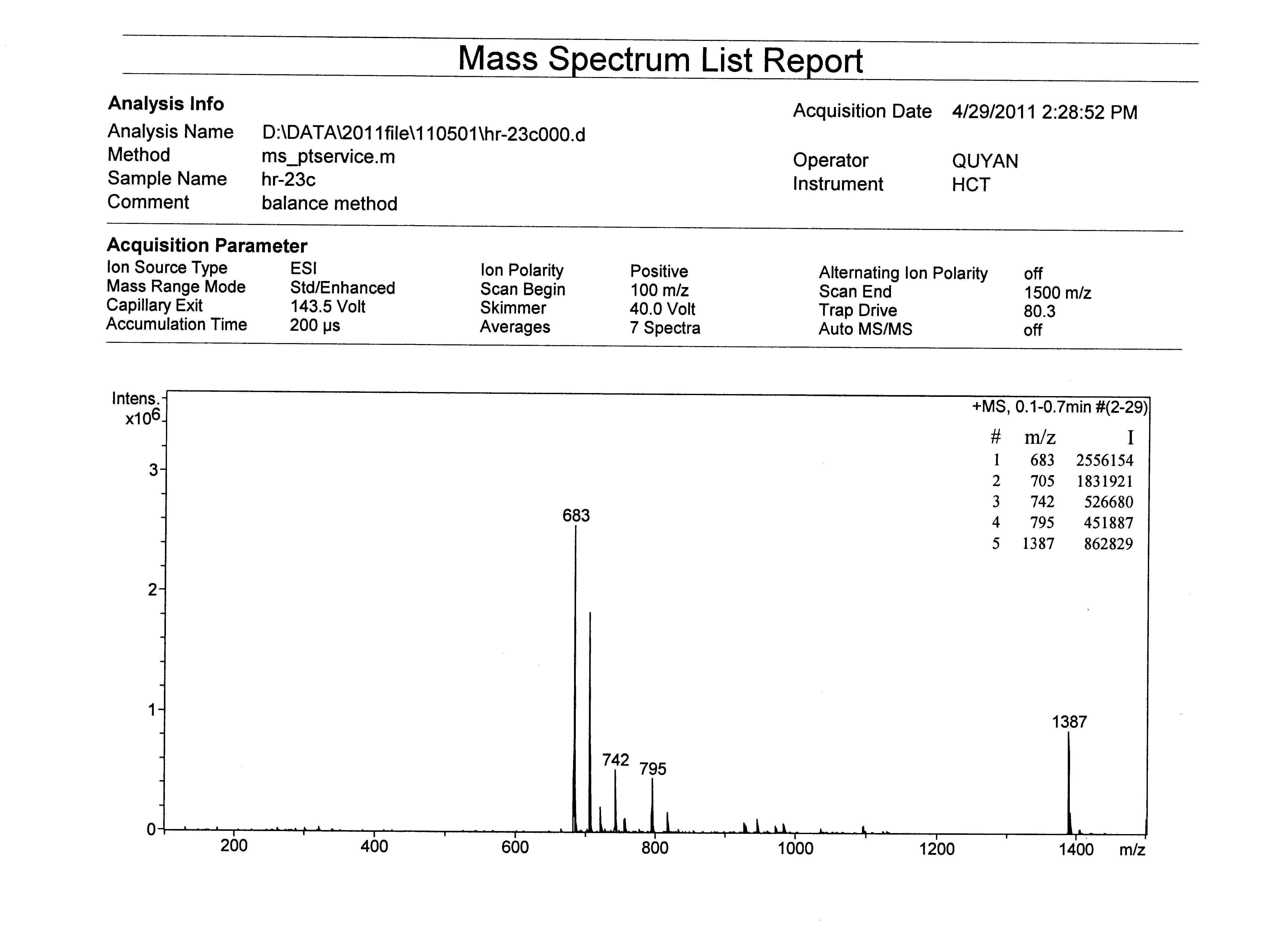


**Figure S6.** HRESI mass spectrum of **1**.


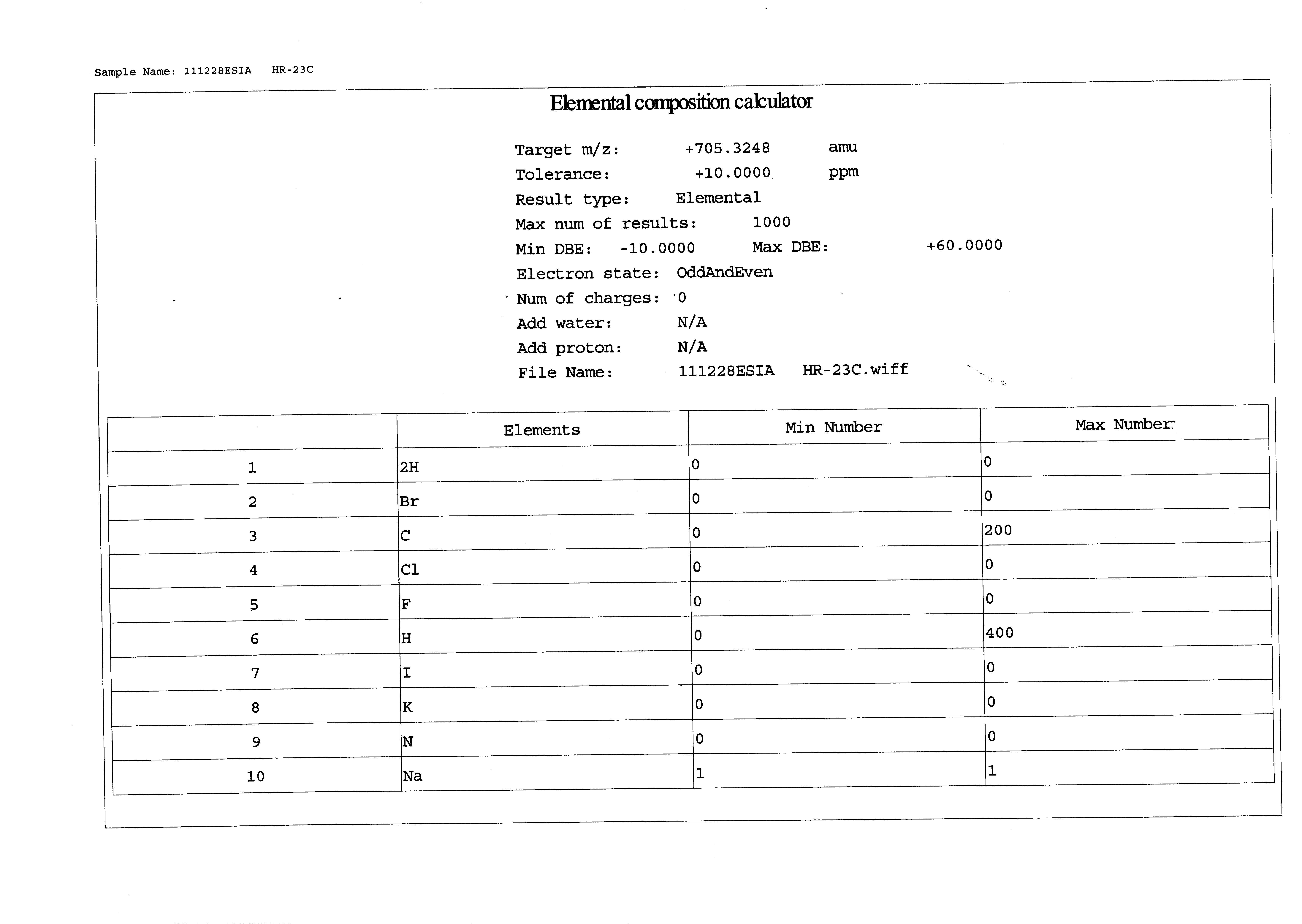

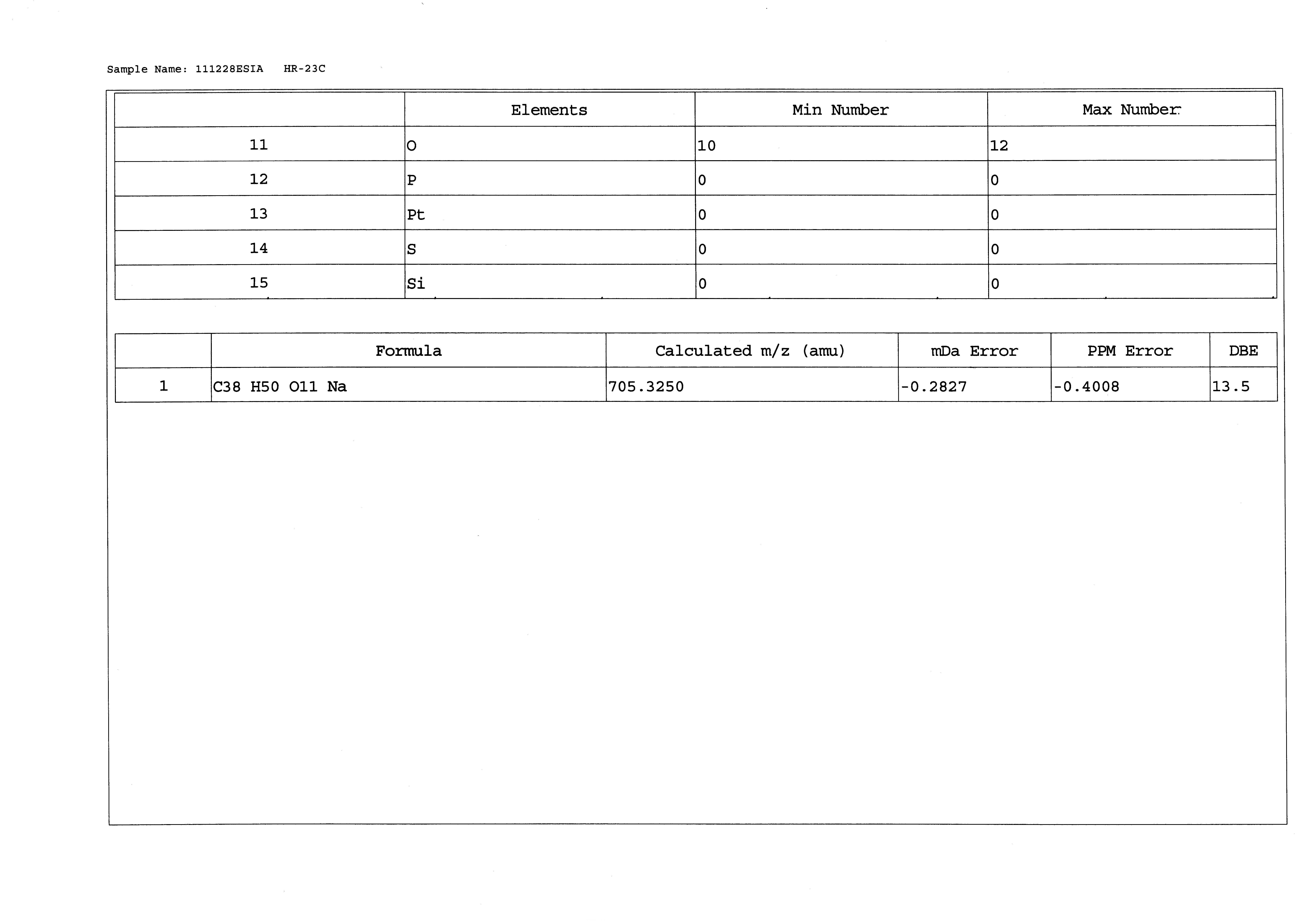


**Figure S7.** ^1^H NMR (400 MHz) spectrum of **1** in CDCl_3_.


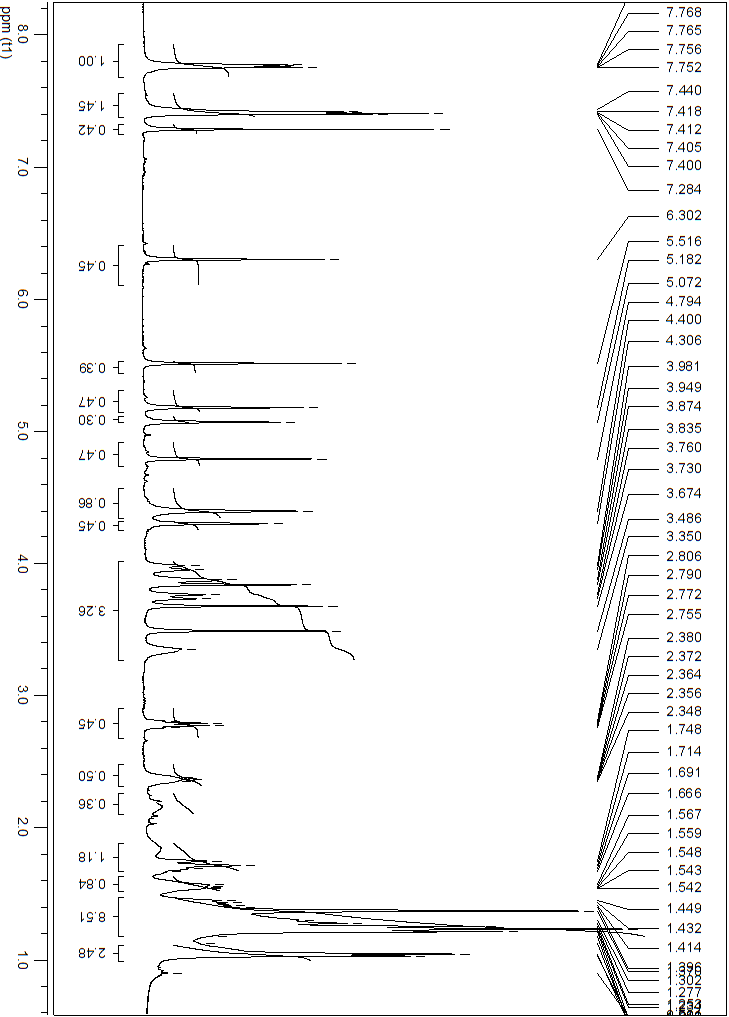


**Figure S8**. ^13^C NMR (100 MHz) spectrum of **1** in CDCl_3_.


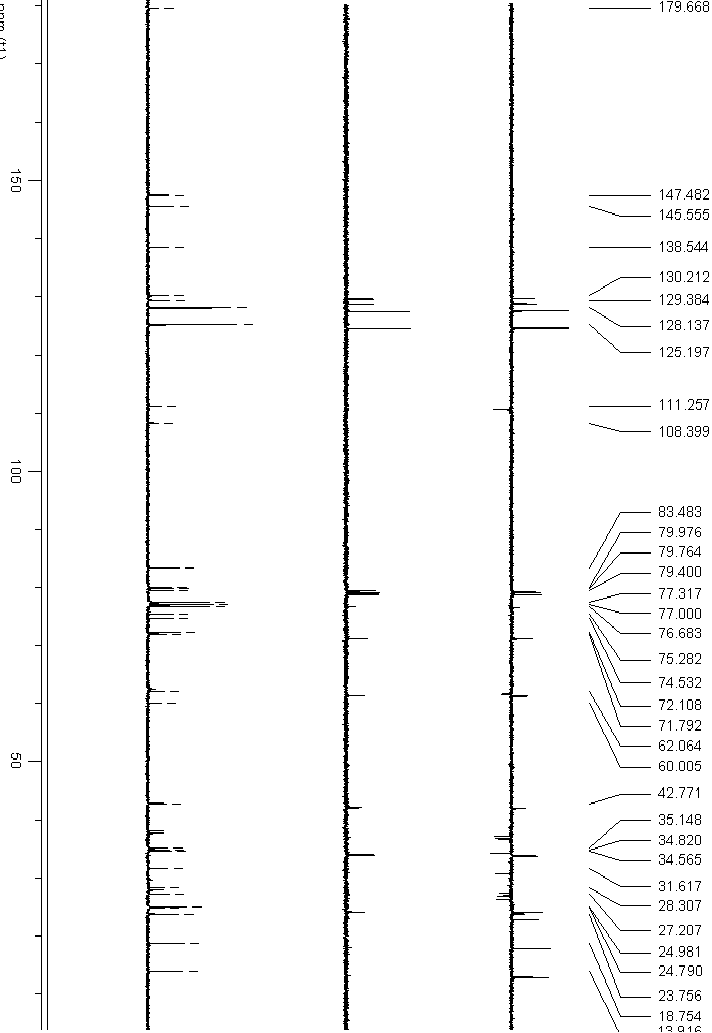


**Figure S9.** HSQC spectrum of **1** in CDCl_3_.


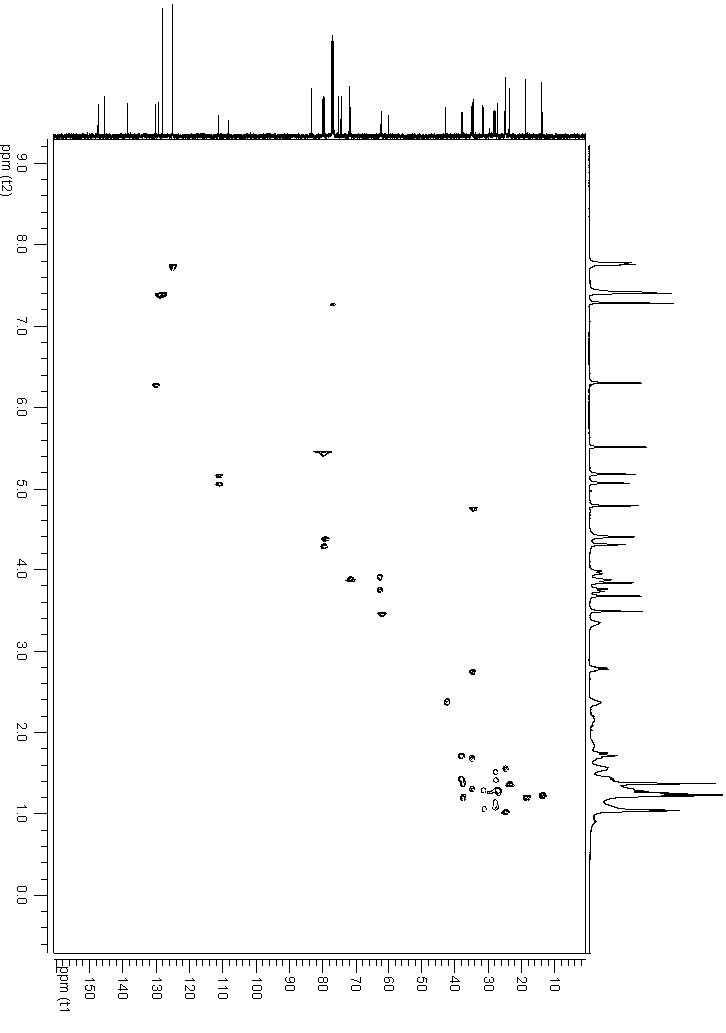


**Figure S10.** ^1^H-^1^H COSY spectrum of **1** in CDCl_3_.


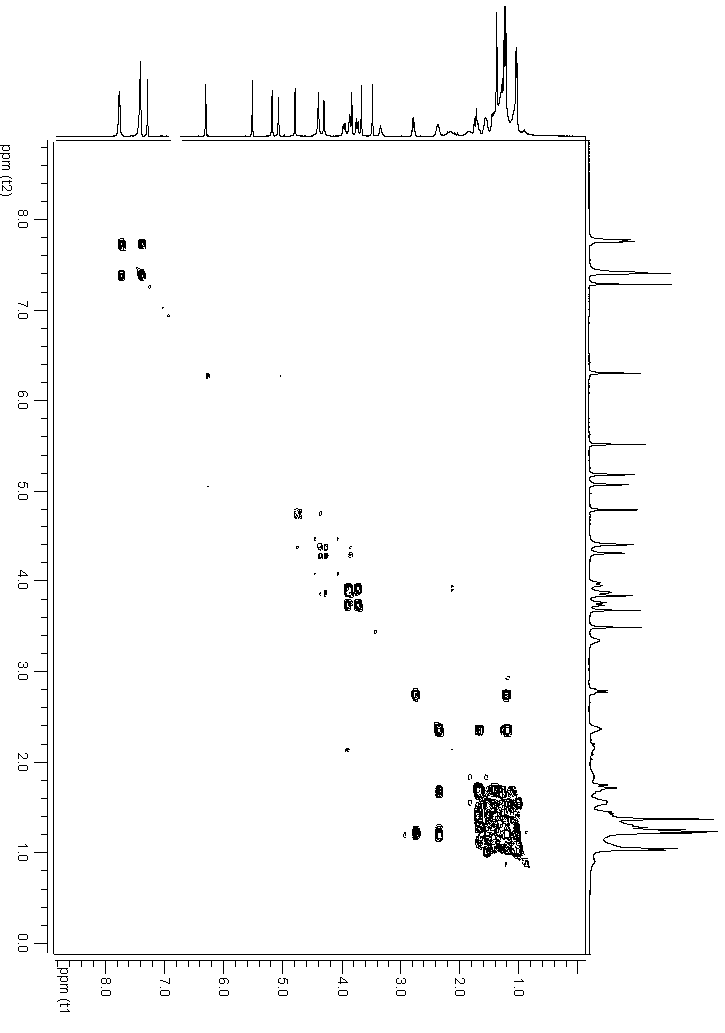


**Figure S11.** HMBC spectrum of **1** in CDCl_3_.


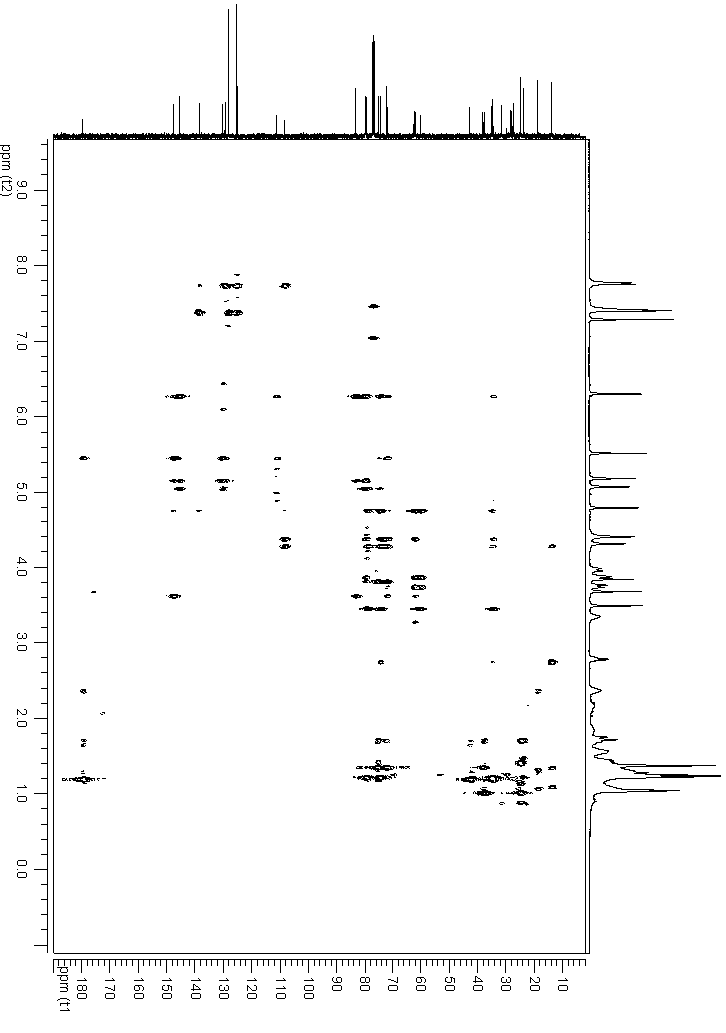


**Figure S12.** ROESY spectrum of **1** in CDCl_3_.


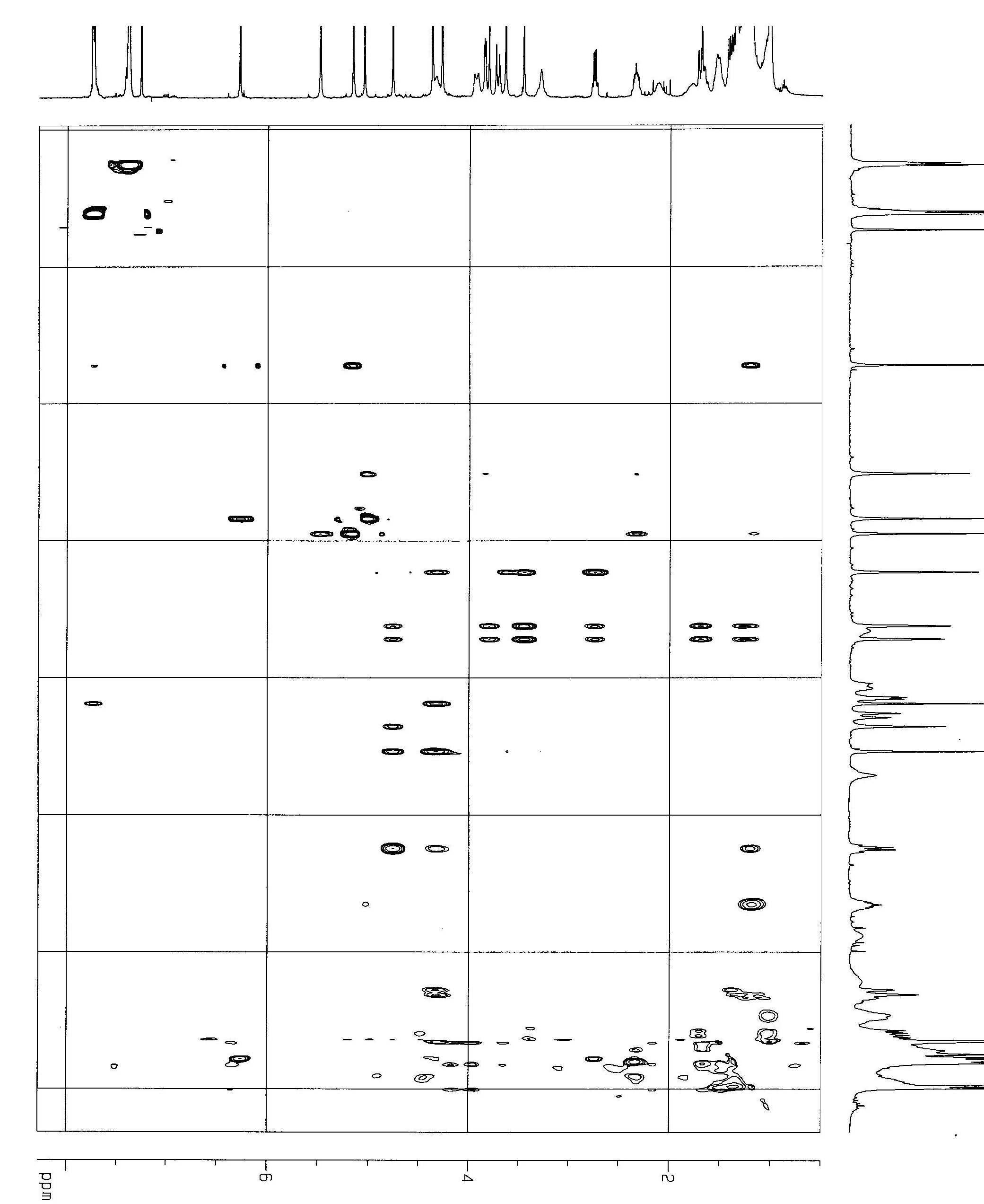


**Figure S13. ESI mass spectrum of 2.**

**
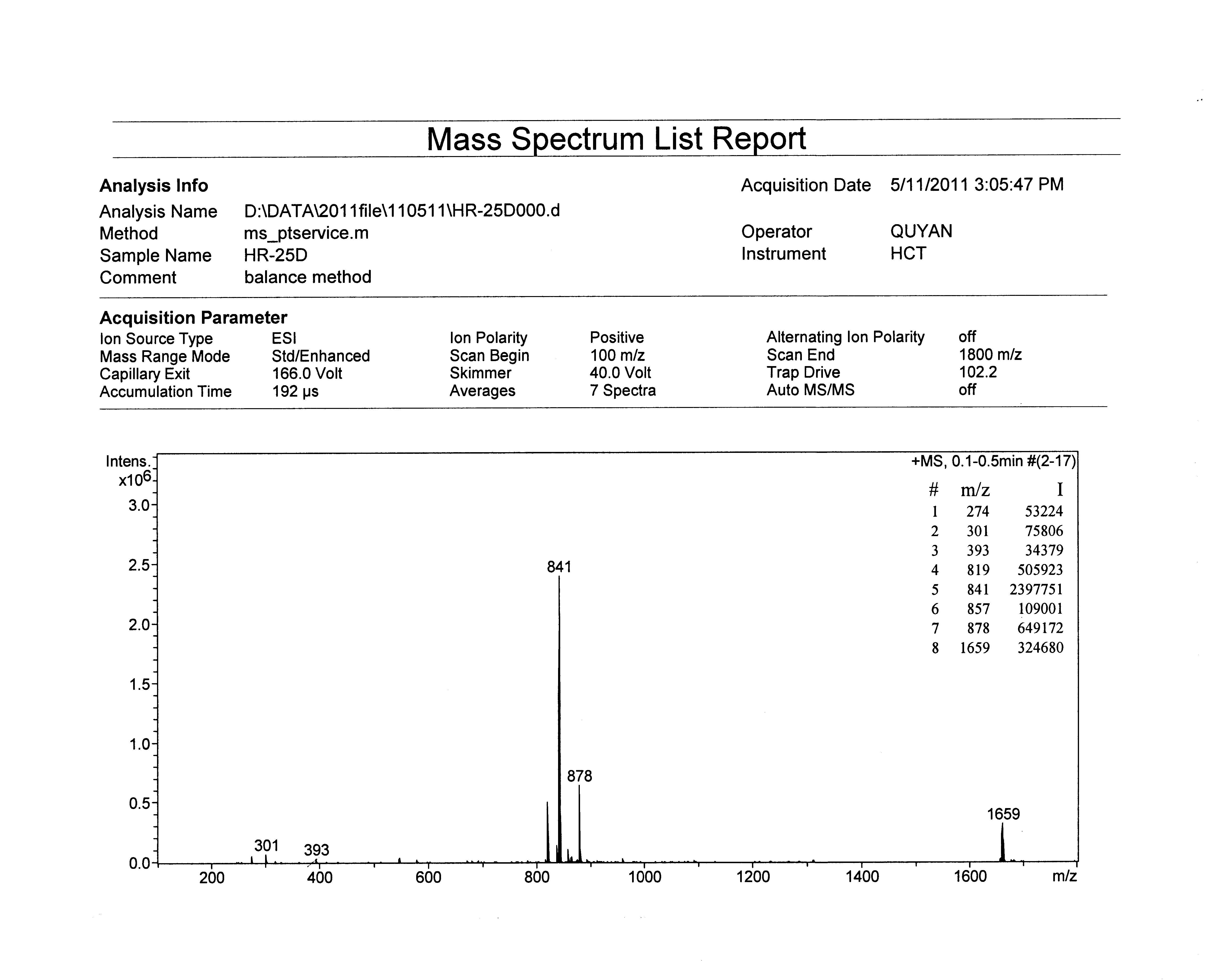
**

**Figure S14. HRESI mass spectrum of 2.**

**
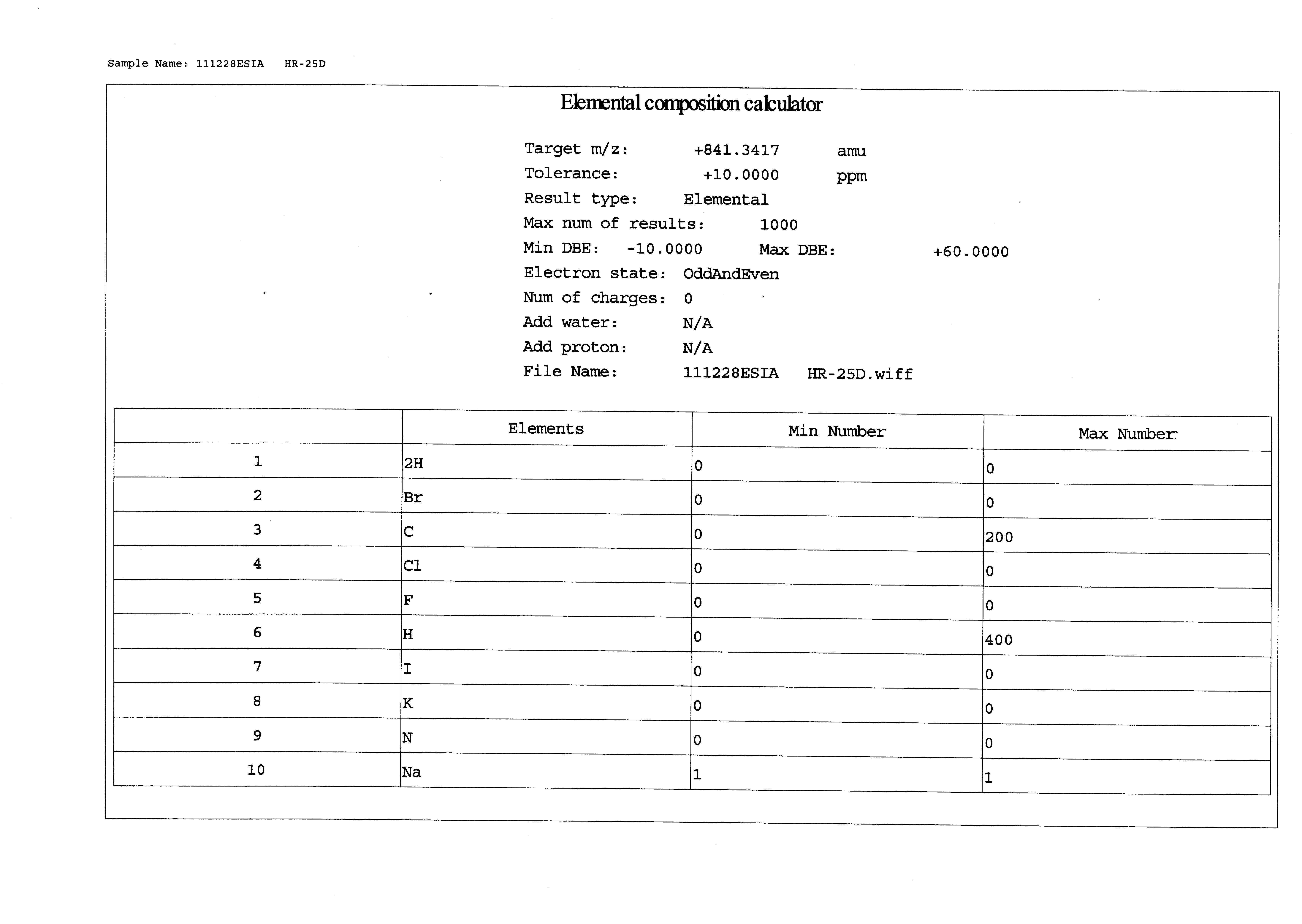

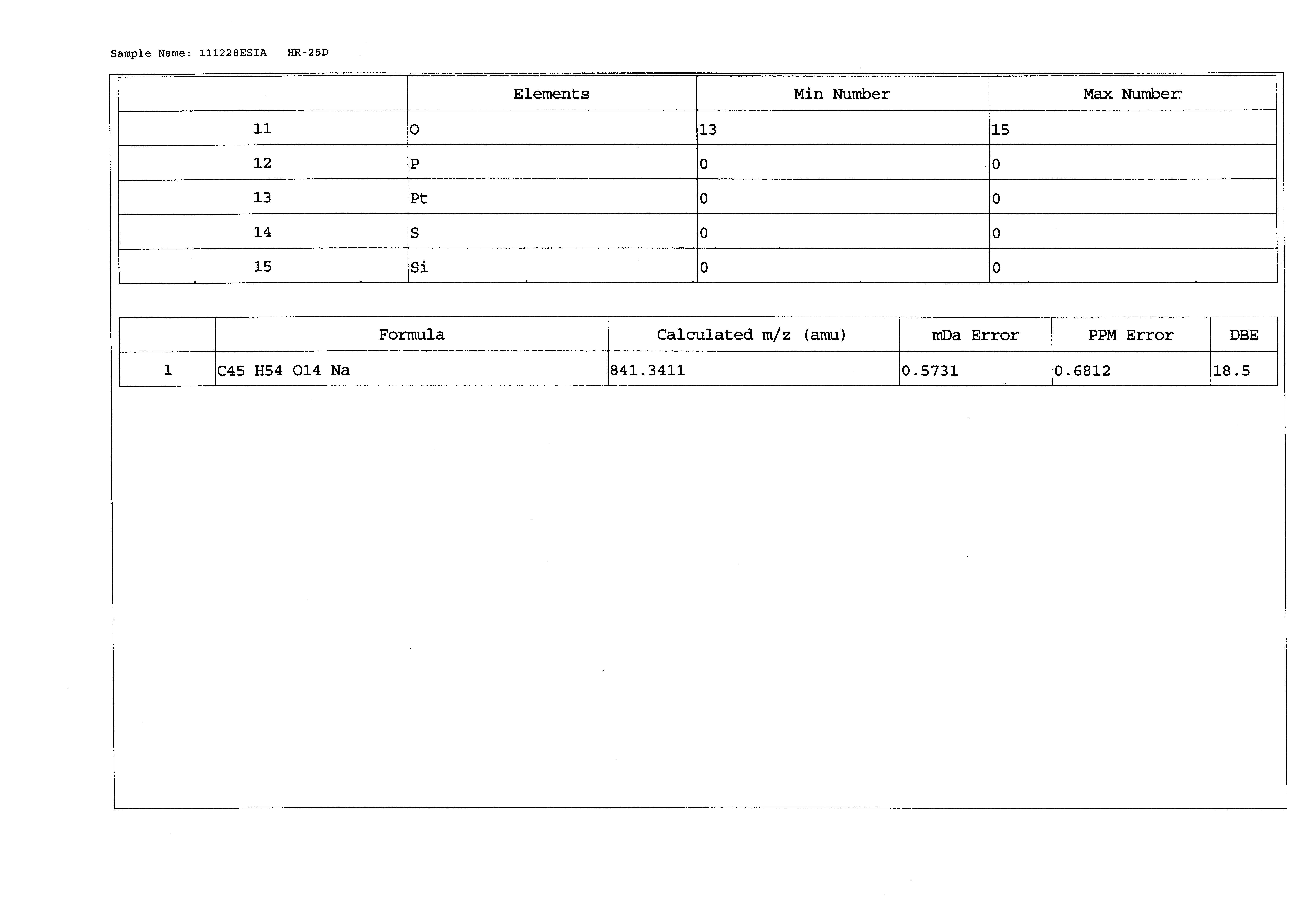
**

**Figure S15. ^1^H NMR (400 MHz) spectrum of 2 in CDCl_3_.**

**
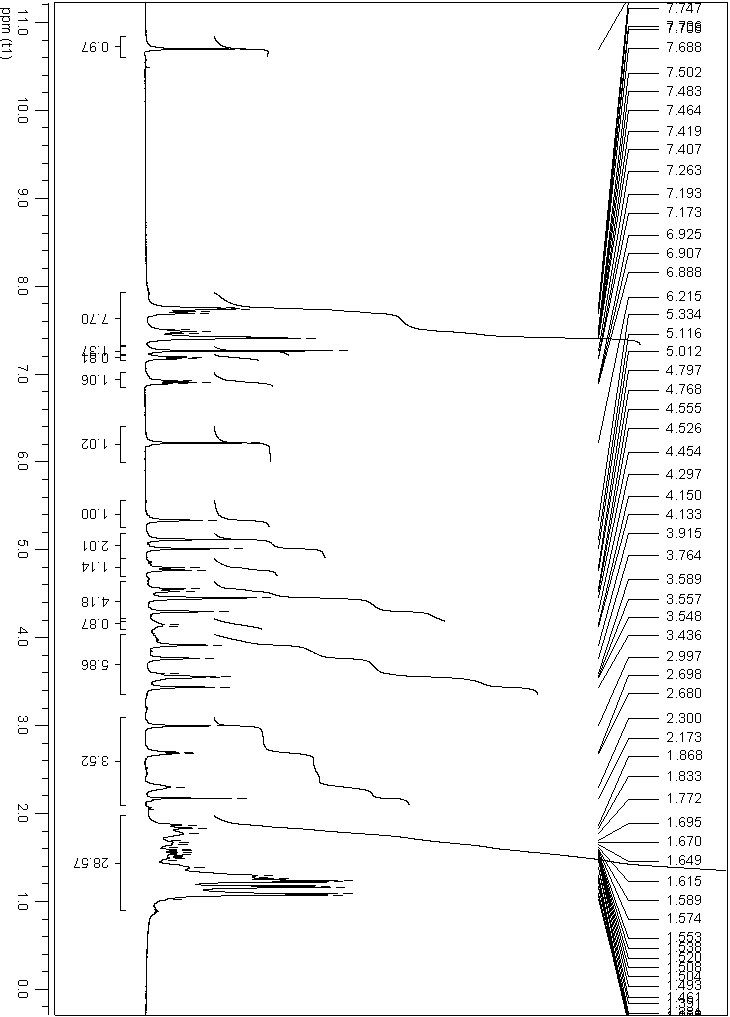
**

**Figure S16. ^13^C NMR (100 MHz) spectrum of 2 in CDCl_3_.**


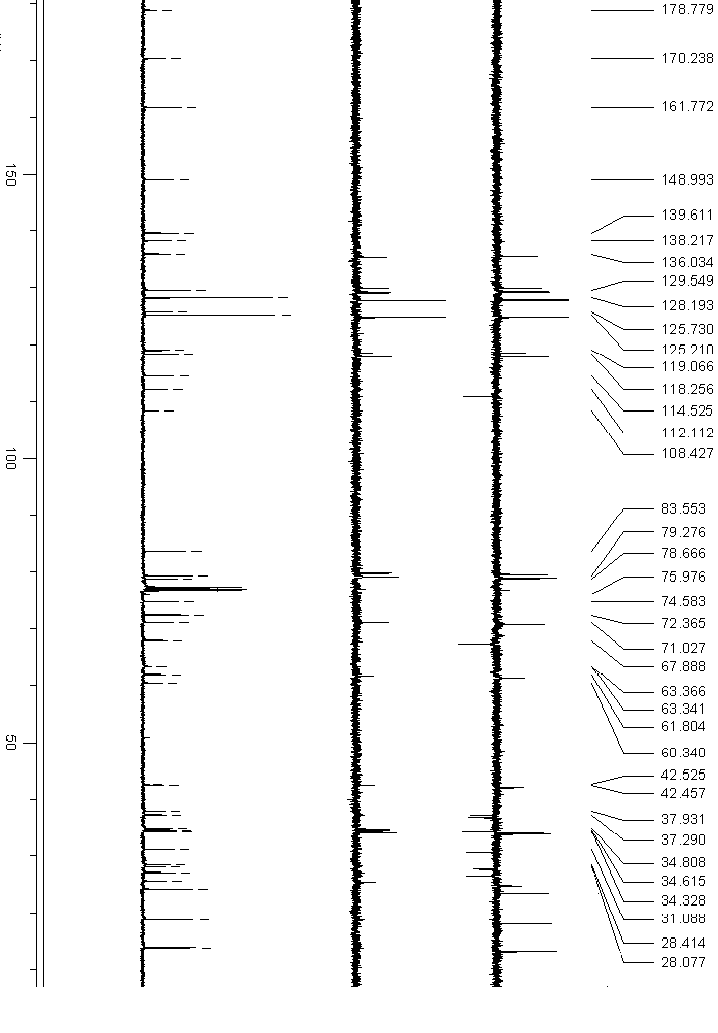


**Figure S17. HSQC spectrum of 2 in CDCl_3_.**

**
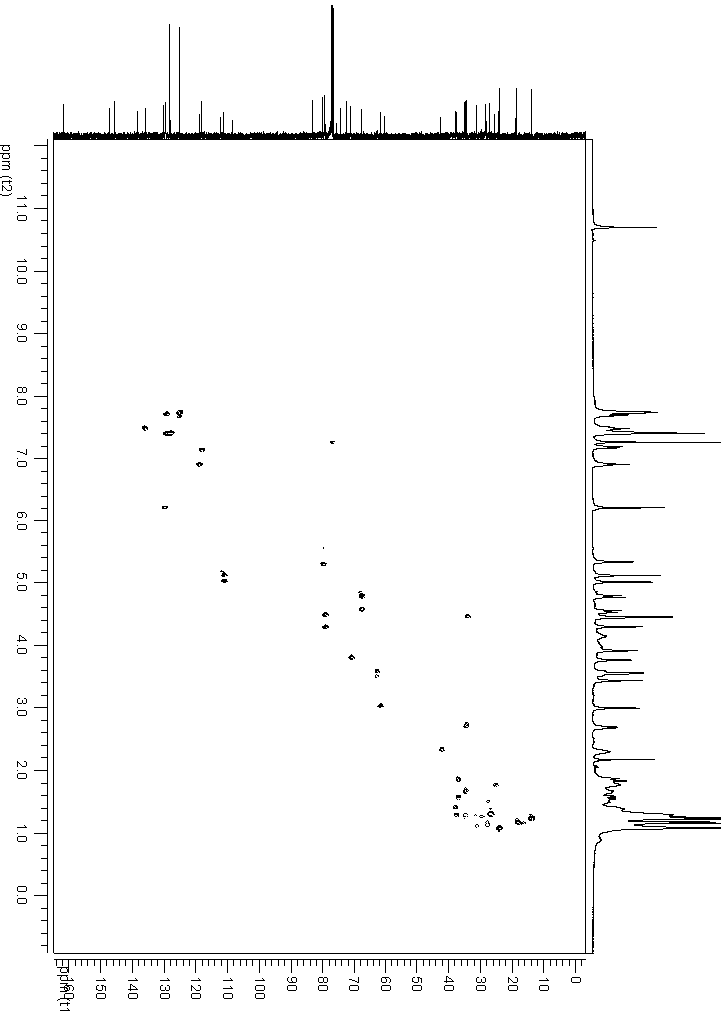
**

**Figure S18. ^1^H-^1^H COSY spectrum of 2 in CDCl_3_.**

**
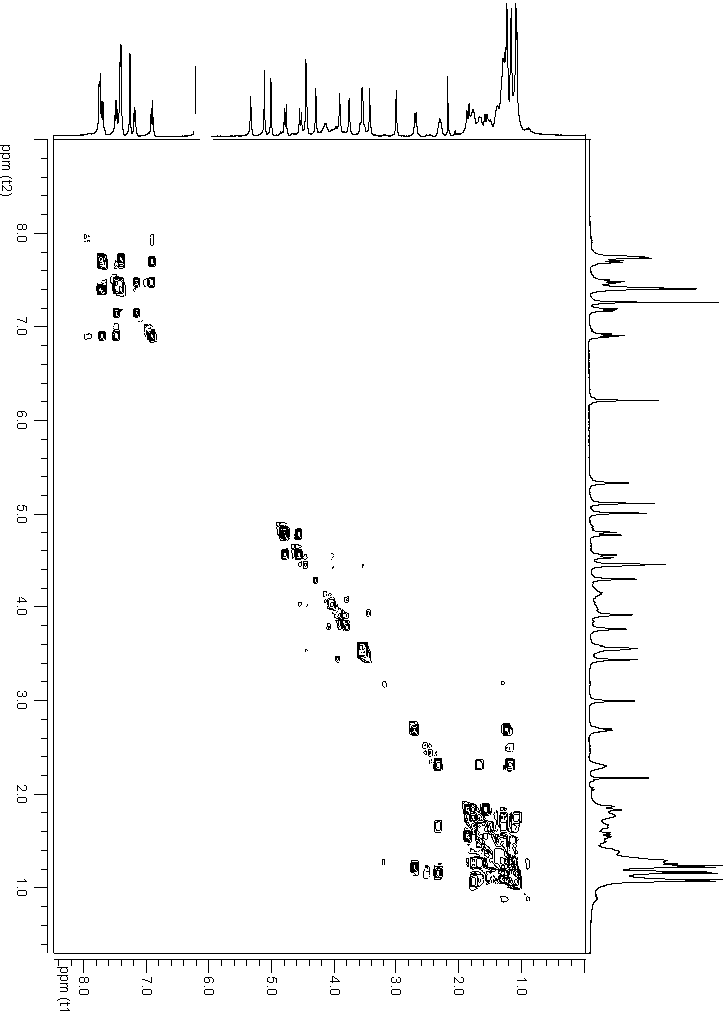
**

**Figure S19. HMBC spectrum of 2 in CDCl_3_.**

**
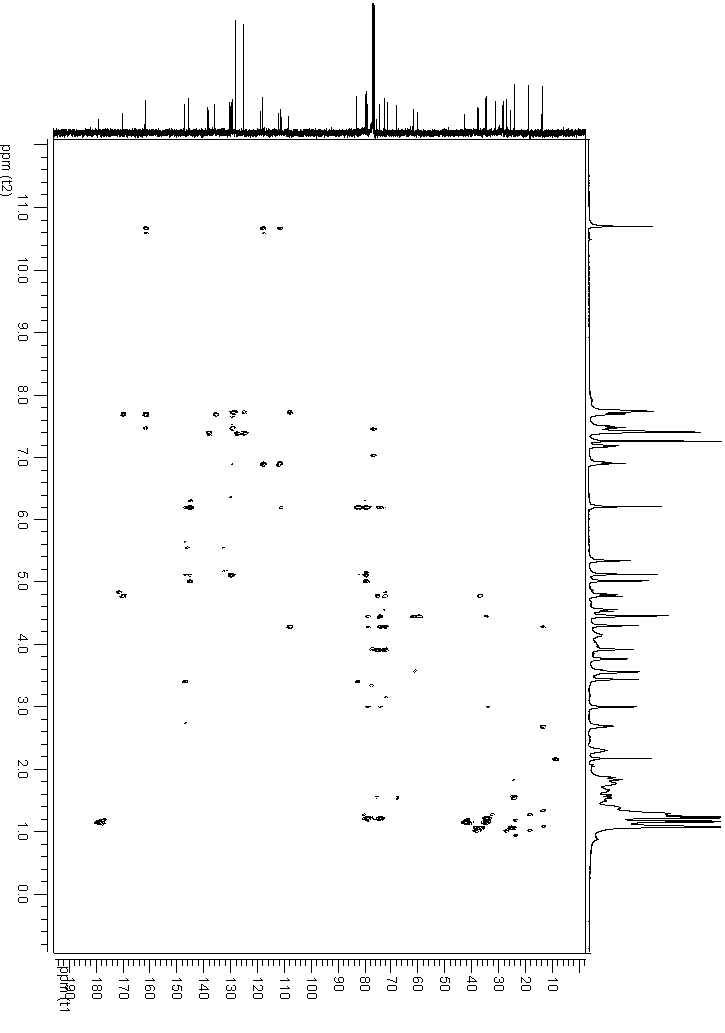
**

**Figure S20. ROESY spectrum of 2 in CDCl_3_.**


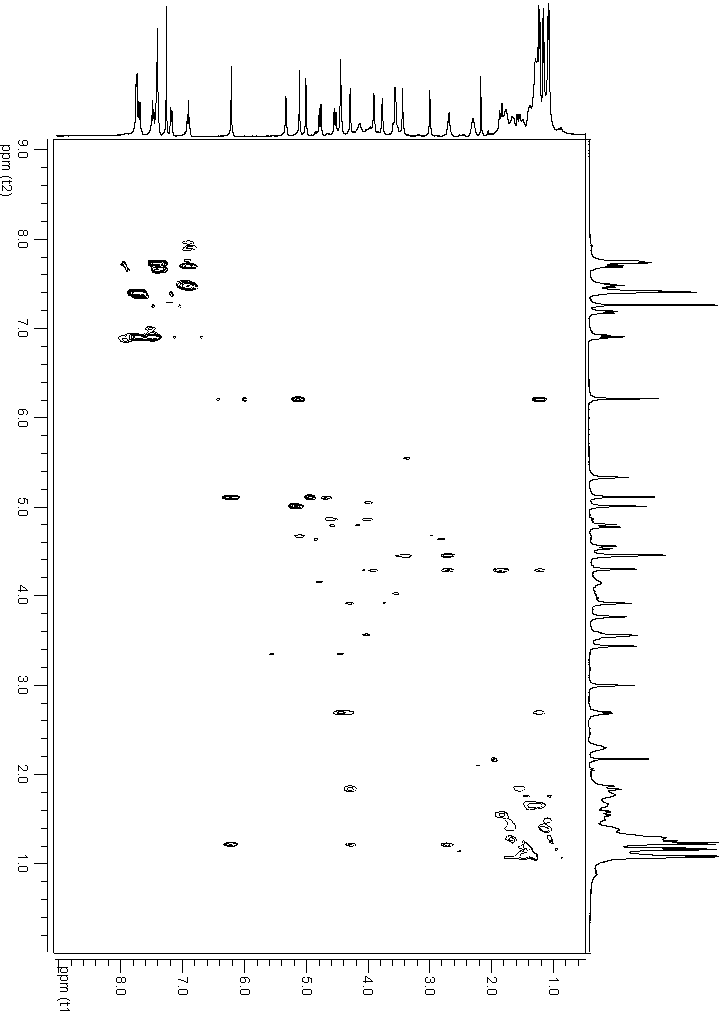


**Figure S21.** ESI mass spectrum of **3**.


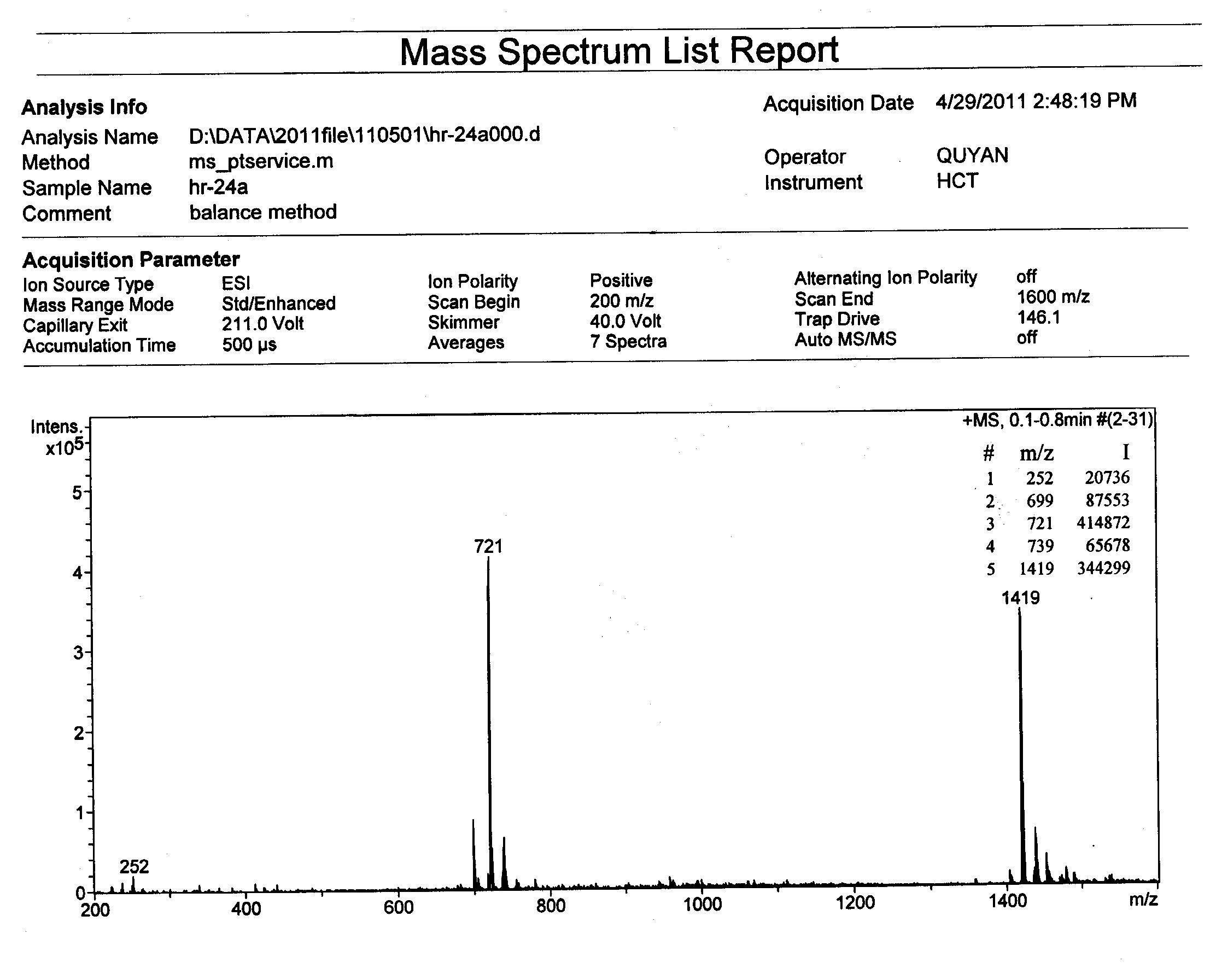


**Figure S22.** HRESI mass spectrum of **3**.


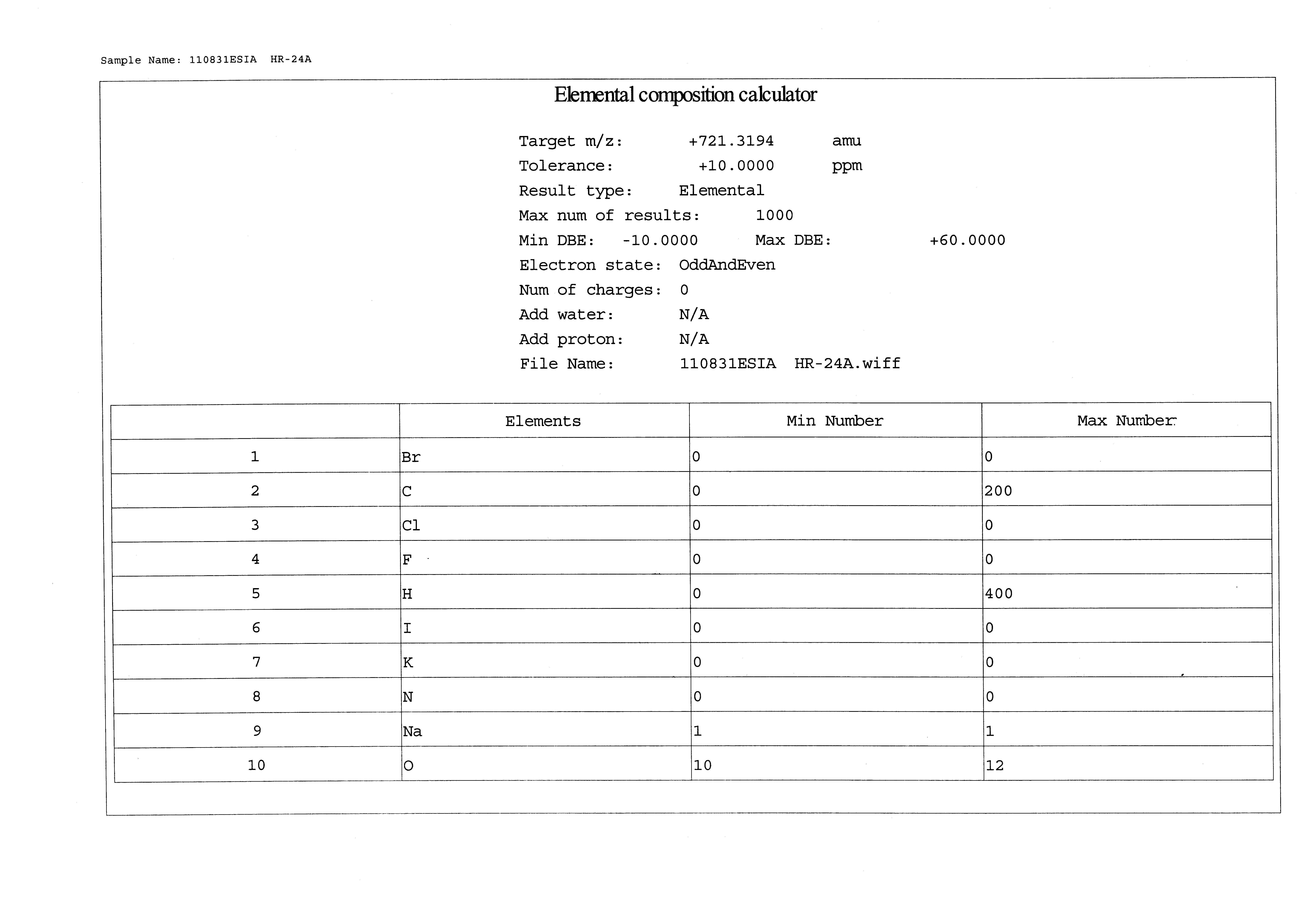

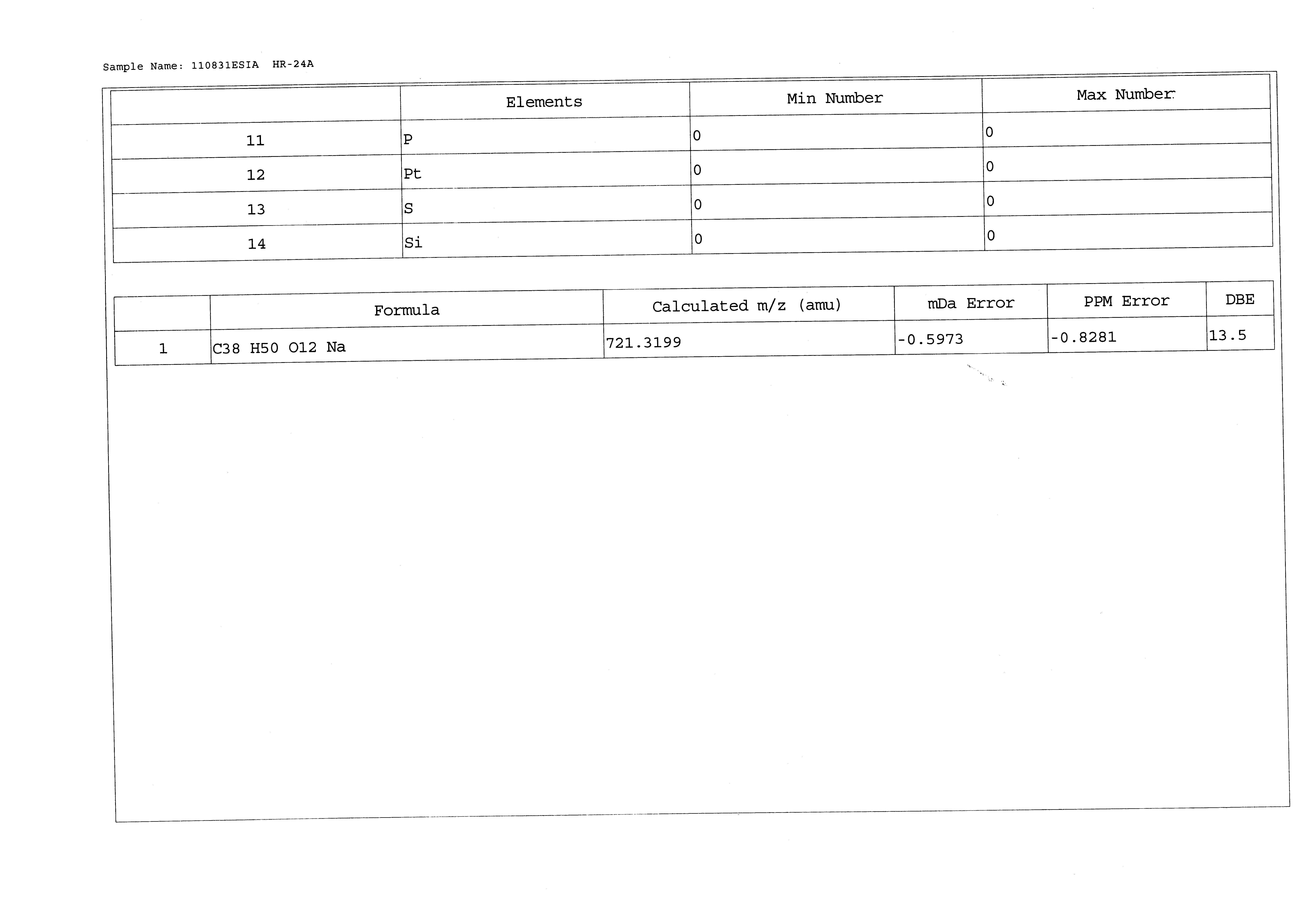


**Figure S23.** ^1^H NMR (500 MHz) spectrum of **3** in CDCl_3_.


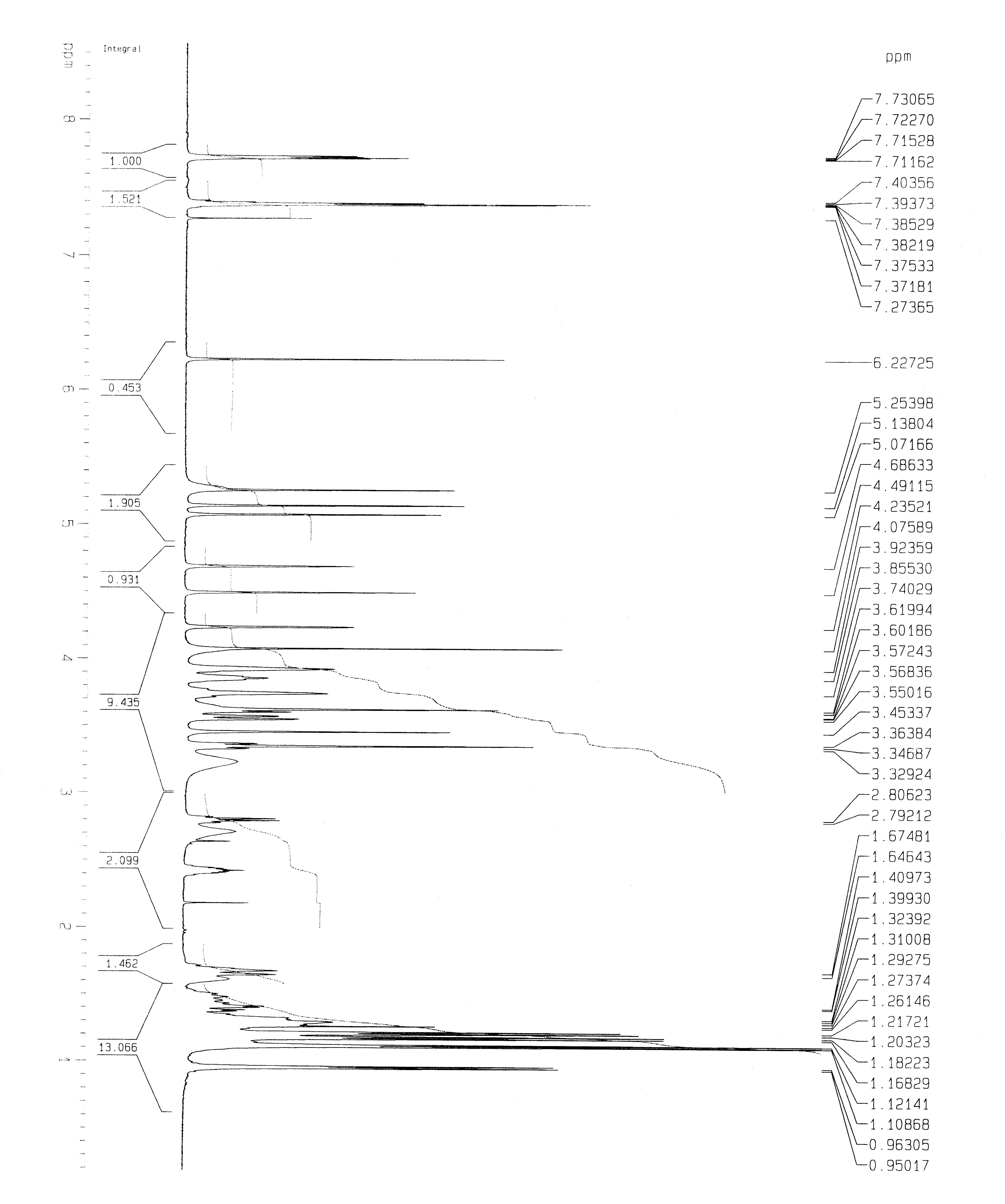


**Figure S24**. ^13^C NMR (125 MHz) spectrum of **3** in CDCl_3_.


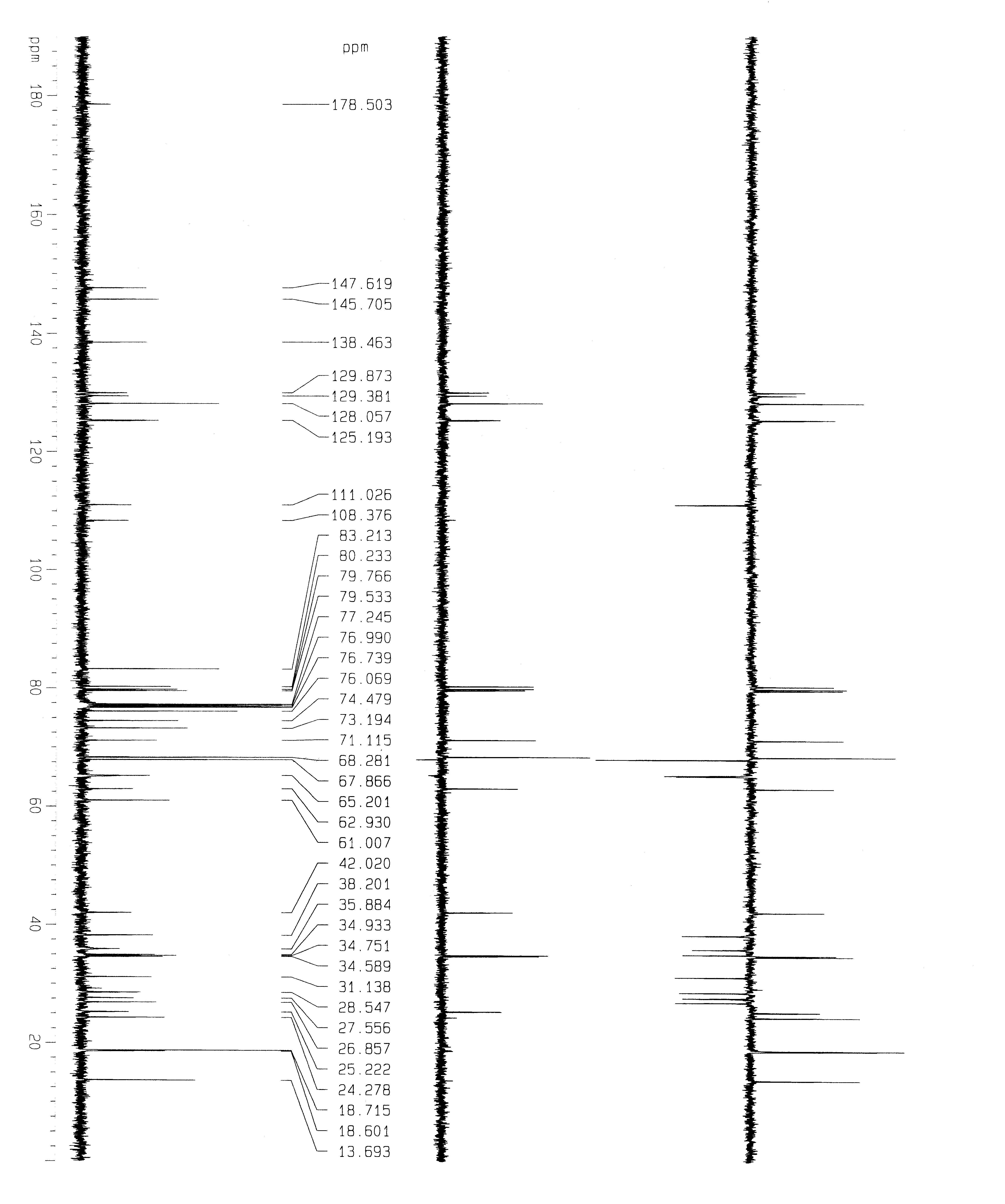


**Figure S25.** HSQC spectrum of **3** in CDCl_3_.


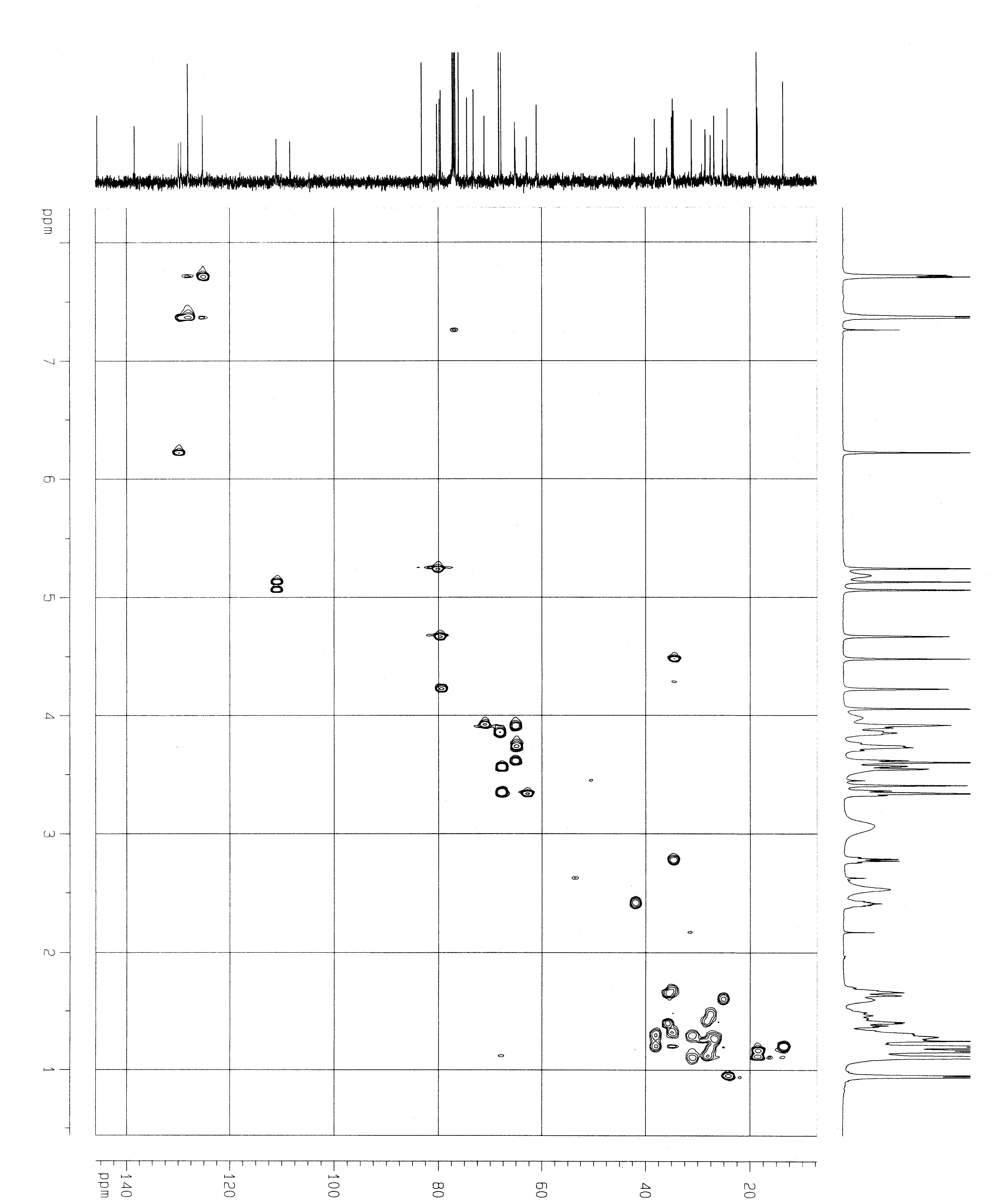


**Figure S26.** ^1^H-^1^H COSY spectrum of **3** in CDCl_3_.


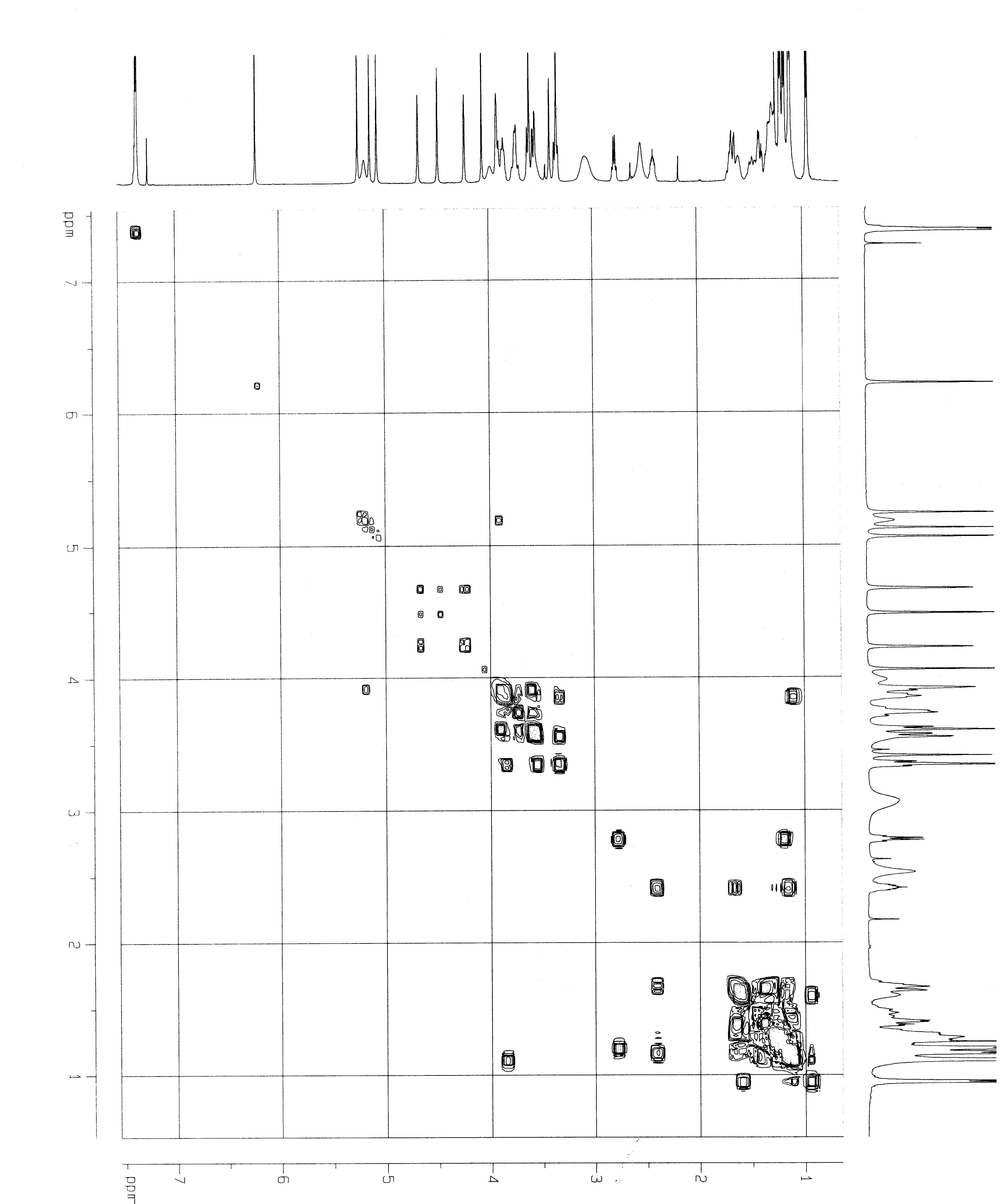


**Figure S27.** HMBC spectrum of **3** in CDCl_3_.


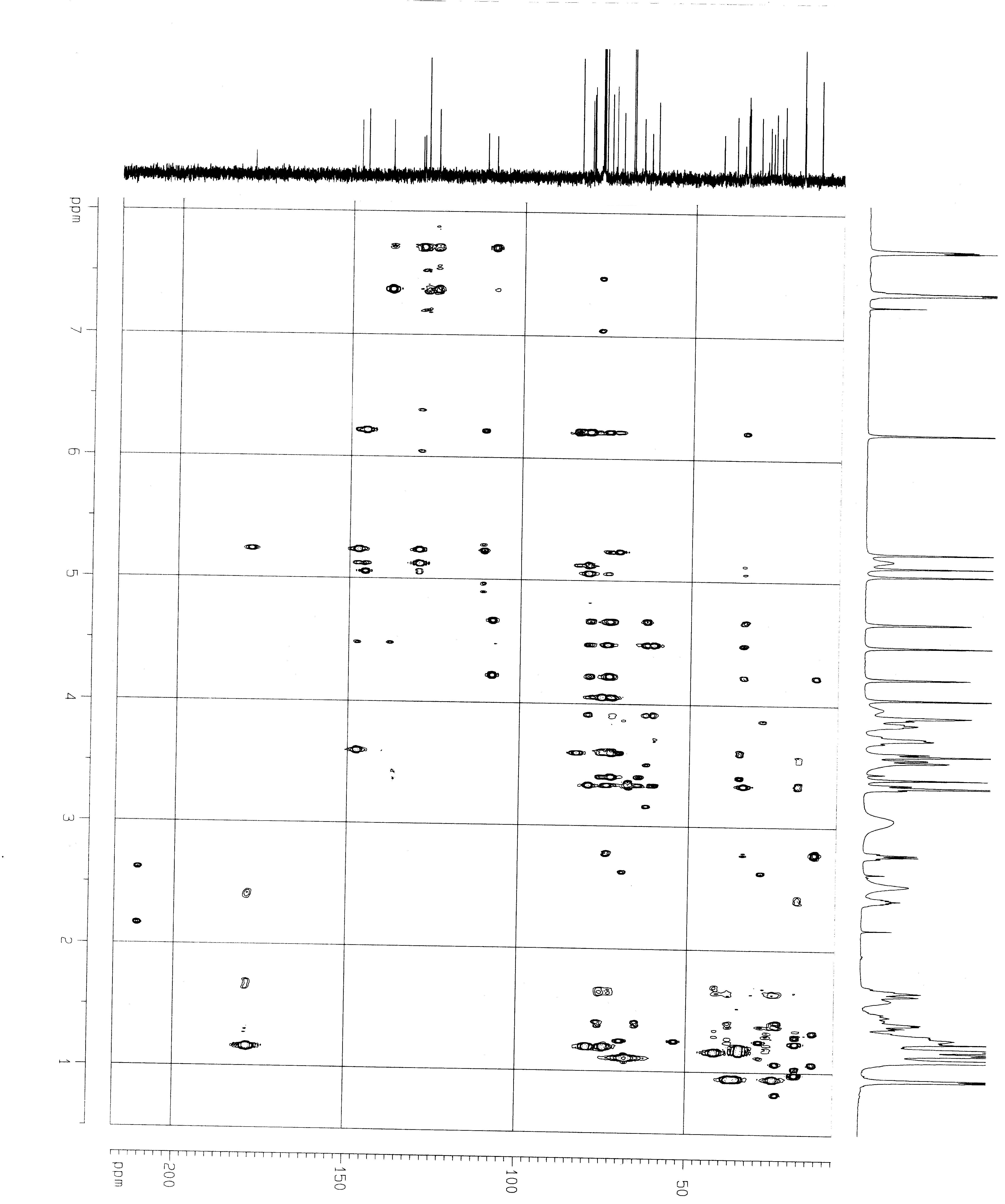


**Figure S28.** ROESY spectrum of **3** in CDCl_3_.


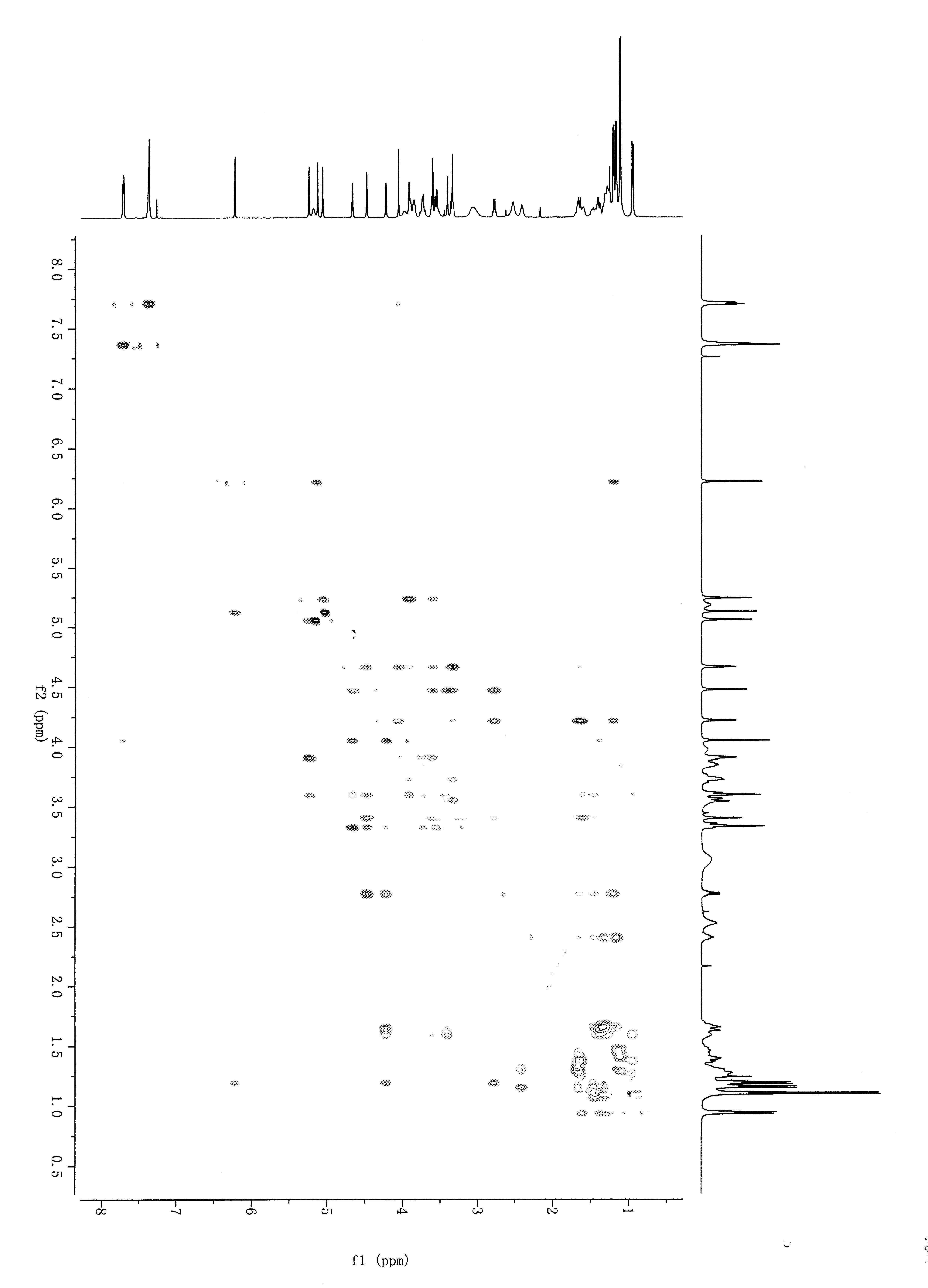

Supplement: Supplementary file 1 — Supplementary file1 Supplementary data (1H and13C NMR data of 1-6; Physical data of 1-6; 1D and 2D NMR of 1-3; Anti-HIV data of 1-6; detailed experimental procedures) can be found. (DOC 16150 kb) [file 13659_2020_231_MOESM1_ESM.doc]
